# Targeting regulated cell death in tumor nanomedicines

**DOI:** 10.7150/thno.67932

**Published:** 2022-01-01

**Authors:** Qinghu Zeng, Xiangyi Ma, Yangmeihui Song, Qiqing Chen, Qiuling Jiao, Liqiang Zhou

**Affiliations:** 1 Department of Ultrasound, The First Affiliated Hospital of Zhengzhou University, Zhengzhou, 450052, China.; 2 Department of Obstetrics and Gynecology, National Clinical Research Center for Obstetrical and Gynecological Diseases, Tongji Hospital, Tongji Medical College, Huazhong University of Science and Technology, 430030, Wuhan, China.; 3 Department of Nuclear Medicine, Union Hospital, Tongji Medical College, Huazhong University of Science and Technology, Wuhan, 430022, China.; 4 Department of Ultrasound, Hainan General Hospital, Haikou, 570311, China.; 5 Cancer Centre and Center of Reproduction, Development and Aging, Faculty of Health Sciences, University of Macau, Macau, SAR 999078, China.; 6 Sino-German Tongji-Caritas Research Center of Ultrasound in Medicine, Department of Medical Ultrasound, Tongji Hospital, Tongji Medical College, Huazhong University of Science and Technology, Wuhan, 430030, China.

**Keywords:** Regulated cell death, Tumor therapy, Nanomedicine, Sensitized apoptosis, Nanomaterials

## Abstract

Nanomedicines hold great potential in anticancer therapy by modulating the biodistribution of nanomaterials and initiating targeted oxidative stress damage, but they are also limited by the inherent self-protection mechanism and the evolutionary treatment resistance of cancer cells. New emerging explorations of regulated cell death (RCD), including processes related to autophagy, ferroptosis, pyroptosis, and necroptosis, substantially contribute to the augmented therapeutic efficiency of tumors by increasing the sensitivity of cancer cells to apoptosis. Herein, paradigmatic studies of RCD-mediated synergistic tumor nanotherapeutics are introduced, such as regulating autophagy-enhanced photodynamic therapy (PDT), targeting ferroptosis-sensitized sonodynamic therapy (SDT), inducing necroptosis-augmented photothermal therapy (PTT), and initiating pyroptosis-collaborative chemodynamic therapy (CDT), and the coordination mechanisms are discussed in detail. Multiangle analyses addressing the present challenges and upcoming prospects of RCD-based nanomedicines have also been highlighted and prospected for their further strengthening and the broadening of their application scope. It is believed that up-and-coming coadjutant therapeutic methodologies based on RCDs will considerably impact precision nanomedicine for cancer.

## Introduction

Conceptually, cellular death can be determined as the perpetual degradation of important cell functions. In line with the newest suggestions of the Nomenclature Committee on Cell Death (NCCD), only cells that display irreparable plasma membrane permeability or undergo thorough disruption are considered dead cells 1. In addition, cases of cellular death can be operationally separated into two reciprocally exclusive regimentations, namely, accidental cell death (ACD) and regulated cell death (RCD). ACD is an immediate and unavoidable cell death induced by extreme physical, chemical or mechanical stimulation and is not sensitive to any form of genetic or pharmacologic intervention 2. In contrast to ACD, RCD touches upon the molecular mechanism of genetic coding and plays an essential role in organismal development, homeostasis, and pathogenesis.

Based on their unique molecular mechanisms and biological aftermath, more than 20 different types of RCD have been broadly classified, including apoptosis, necroptosis, pyroptosis, entosis, ferroptosis, etc. 2. As a cell suicide pathway, RCD can be initialized if the adaptive response to disturbance of the cellular microenvironment fails. To a certain extent, RCD can also be regulated by restraining the transduction of fatal signals and augmenting the ability of cells to respond adaptively to stress based on genetic manipulations or drugs 3, 4. Various antitumor therapies use the destructive potential of cell suicide pathways to destroy cancer cells, emphasizing the crucial significance of RCD in combating cancer. The development of nanotechnology in the field of biomedicine has made tremendous progress in recent years, and various nanomaterials have been widely utilized in tumor treatment due to their own unique advantages in targeting tumors and delivering therapeutic agents 5. However, this single apoptosis-inducing effect cannot effectively inhibit tumor progression owing to the apoptosis resistance machinery of certain cancer cells.

**Figure 1:**
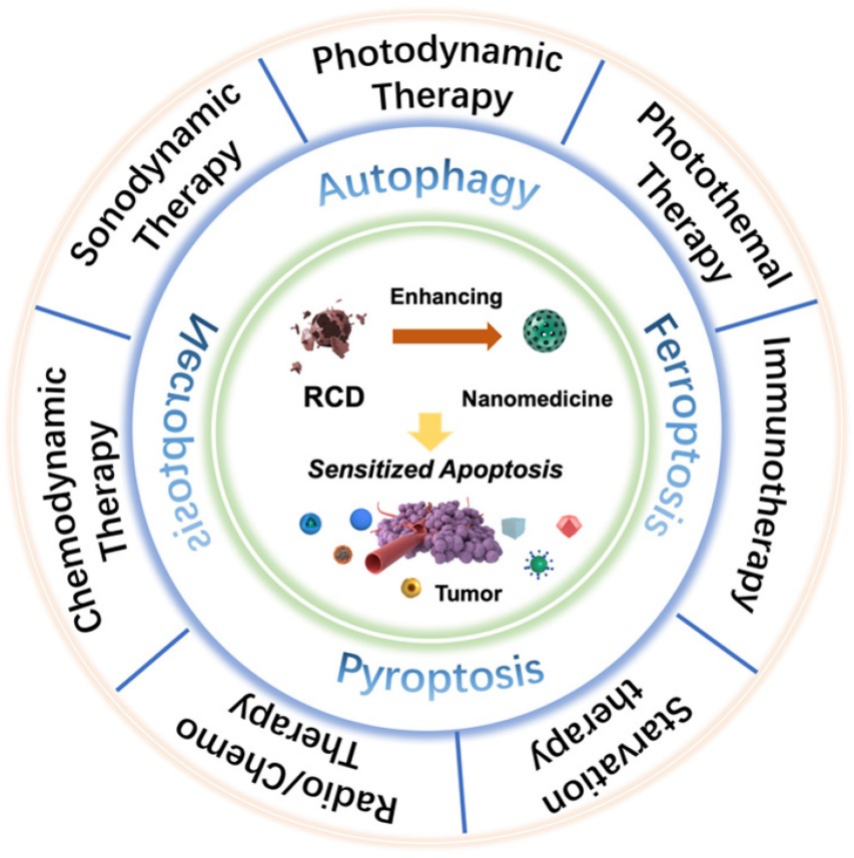
Scheme of regulated cell death (RCD)-sensitized synergistic cancer nanomedicines based on various nanomaterials.

An increasing number of studies have found that rationally designed nanomedicines can remarkably improve the therapeutic effect of tumors by triggering or inhibiting RCD, which may lead to novel ideas for future cancer therapies. Here, we concentrate on RCD-based collaboratively improved cancer nanotherapy. First, we provide an overview of various types of RCD, including autophagy, ferroptosis, pyroptosis, and necroptosis, and their application strategies in the field of nanomedicine. Second, multiple tumor nanotherapy applications combined with RCD are outlined in detail, such as photodynamic therapy (PDT), sonodynamic therapy (SDT), photothermal therapy (PTT), immunotherapy, chemodynamic therapy (CDT), starvation therapy, radiotherapy and chemotherapy (**Figure 1**). Finally, current challenges and the future outlook of RCD-based nanomedicines will be discussed. Because of the latent auxiliary mechanism of RCD and reasonably designed multifunctional nanobiomaterials, it is believed that such cooperative therapeutic combinations will pave the way for new and advantageous interventions to sensitize refractory tumors.

## Overview of RCD-based Strategies

### Autophagy

As a lysosome-mediated intracellular biological degradation process, autophagy can be divided into five consecutive processes: autophagic initiation, autophagic nucleation, autophagic elongation, autophagic fusion, and autophagic degradation 6. Many steps in the process of autophagy exist as latent therapeutic targets and offer several methods of eliciting positive and negative effects on autophagy. The adaptor protein sequestosome 1 (p62), which targets special substrates to autophagosomes, and the autophagosome marker protein microtubule-associated protein 1 light chain 3II (LC3II) can be degraded together with other cargo proteins, thus being applied as a representative marker of autophagic flux 7. Successfully targeted autophagy will significantly augment the efficiency and effect of tumor nanotherapy.

Generally, basal autophagy can not only clear damaged cellular organelles, proteins and pathogens to maintain organism homeostasis but also prevent genome instability to promote cell survival 8, 9. Various pathological and pathophysiological conditions, such as physical, chemical, or biological stress-induced autophagy, may result in two distinct cell fate paradigms: pro-survival or pro-death 10, 11. Current efforts to inhibit or induce autophagy have been reported and have been shown to cause prompt tumor cell death or to restrain tumor growth. Therefore, it is necessary to discuss and deploy numerous nanomedicine application strategies based on the different cell fate results of autophagy to enhance the effect of tumor treatment.

### Ferroptosis

Ferroptosis is an iron-dependent RCD form involving lethal, iron-catalyzed lipid peroxidation damage, which represents an inherent vulnerability induced by the combination of polyunsaturated fatty acids into cell membranes 12. The sensitivity of ferroptosis is closely associated with multiple biological processes, such as glutathione, nicotinamide adenine dinucleotide phosphate (NADPH), and phospholipid biosynthesis, as well as the metabolism of polyunsaturated fatty acids, amino acids, and iron 13. There are numerous ways to achieve the induction and modulation of ferroptosis, such as the depletion of intracellular glutathione (GSH) 14, the inactivation of glutathione peroxidase 4 (GPX4) 15, and the excessive delivery of iron into cancer cells 16.

To enable smooth growth, cancer cells frequently display an improved iron demand, and this iron dependency will make cancer cells more vulnerable to ferroptosis 17, 18. Ferroptosis therefore exhibits a predominant tumor-suppressor function and is being explored as an alternative way to eliminate cancer cells, especially for clones with acquired or intrinsic resistance to apoptosis 19. Compelling evidence has also demonstrated the anticancer potential of targeting ferroptosis based on the vulnerability of apoptosis-resistant cancers to ferroptosis. Some iron-containing nanoparticles can release intracellular iron under the stimulation of the acidic microenvironment of cancer cells, thereby enhancing Fenton-like reactions and reactive oxygen species (ROS) production and ultimately killing cancer cells 20. The full utilization of nanomedicines to trigger dual ferroptosis/apoptosis may be a rising anticancer strategy.

### Pyroptosis

Pyroptosis is an inflammatory form of cell death caused by some inflammasomes that results in the cleavage of gasdermin D (GSDMD) and the activation of inflammatory cytokines such as interleukin-1beta (IL-1β) and interleukin-18 (IL-18) 21, 22. Lysed GSDMD releases the gasdermin-N domain (N-GSDMD), which transfers to the cell membrane and promotes the formation of membrane pores (GSDMD pores), ultimately inducing cell swelling, membrane breakdown and cell death 23, 24. In addition, the released cellular components, including lactate dehydrogenase (LDH) and inflammatory cytokines, can play an auxiliary antitumor effect by priming the antitumor immune response 25.

As a prospective way to fight against cancer, regulating the pyroptosis death pathway can effectively inhibit the proliferation and migration of cancer cells. Generally, pyroptosis can be triggered by chemodrug-induced ROS, but drug resistance and side effects severely restrict its biomedical applications 26, 27. An increasing number of studies have focused on more effective and noninvasive methodologies to activate pyroptosis based on advanced nanotechnologies, such as PDT 28, nanocatalytic therapy 29, and PTT 30. There is an urgent need to explore more powerful tumor treatment approaches by utilizing pyroptosis-based cancer therapy.

### Necroptosis

As a kind of caspase-independent programmed necrosis, necroptosis appears to be a novel therapeutic target, causing dying cancer cells to stimulate the immune response 31. In contrast to the poor immunogenicity of apoptosis, necroptosis more efficiently boosts antitumor immunity due to the strong immunogenicity of proinflammatory molecules 32, 33. Receptor-interacting protein kinase 3 (RIPK3) plays a key role in the process of necroptosis, which can effectively activate mixed-lineage kinase domain-like protein (MLKL) and promote membrane disintegration 34, 35. Typical morphological features of necroptosis include increased organelle volume, decreased plasma membrane integrity, and the leakage of intracellular components 36.

Induction and/or manipulation of necroptosis in anticancer therapies represent a prospective therapeutic approach to overcome inherent tumor resistance to apoptosis, serving as an alternative method to eradicate apoptotic-resistant cancer cells. Subsequent studies have indicated that radiotherapy and chemotherapy could trigger necroptotic cell death 37, 38. Additionally, a series of metal nanoparticles, such as silver (Ag) nanoparticles 39, selenium (Se) nanoparticles 40, and zinc oxide (ZnO) nanoparticles 41, have also been found to trigger ROS-mediated necroptosis in cancer cells. More nanotechnologies urgently need to be explored and developed to target necroptosis.

## Synergistic Tumor Nanotherapy based RCD

### Photodynamic Therapy

PDT is a clinically available and approved therapeutic option for certain types of cancers that have emerged as an alternative or additional methodology to traditional radio/chemotherapy 42. Its advantages include its noninvasiveness, spatiotemporal controllability, and few side effects. The chemical mechanism of PDT involves the delivery of photosensitizers to the tumor site, followed by irradiation with a particular wavelength of light to generate deleterious cytotoxic ROS to initiate a photochemical oxidative stress reaction 43. Apoptosis is considered to be the definite molecular mechanism of PDT toxicity induction, generally manifested by cell contraction and nuclear fragmentation 44, 45. However, the effectiveness of PDT for apoptosis induction is limited owing to the acquired or intrinsic resistance of cancer cells to apoptosis 46. Therefore, exploring and manipulating other forms of RCD triggered by PDT opens up avenues for new therapeutic approaches to eliminate cancer cells and to limit apoptosis-resistant variants.

#### Regulated autophagy-enhanced PDT

In the process of PDT, the crosstalk between apoptosis (“self-killing”) and autophagy (“self-eating”) may have an eventful influence on the suppression efficacy of tumors. Self-protective autophagy generally plays a role in promoting survival by recycling nutrients to cancer cells 47. To overcome autophagy-induced resistance of cancer cells to ROS damage, numerous studies have proposed a synergistic tumor suppression strategy of PDT and autophagy inhibition. Yu *et al.* designed and synthesized bovine serum albumin (BSA)-modified zinc phthalocyanine (BSA-ZnPc, BZ) nanoparticles to achieve enhanced antitumor effects based on BZ-induced PDT and 3-methyladenine (3-MA)-induced autophagy inhibition 48. Since PDT-induced autophagy can lead to compromised cell killing effects, autophagy suppression during PDT is a collaborative strategy worth exploring. It was demonstrated that combinational therapy remarkably improved PDT-mediated anticancer efficiency in osteosarcoma (OS) cells via activation of the caspase-independent signaling pathway, resulting in a cell killing rate of more than 90% at a concentration of 2.5 μg/mL 49. 3-MA blocks the formation of autophagosomes in the early stage of PDT-induced cytoprotective autophagy, which makes tumor cells unable to recycle damaged nutrients. In addition, an *in vivo* study combining 3-MA with PDT showed that the volume of bilateral orthotopic xenograft tumors was significantly smaller after two intravenous injections of BZ nanoparticles and light radiation in the right flank, indicating immunotoxicity to distant tumor metastasis of synergistic therapy. However, overactive autophagy may generate the opposite results and transform the role of autophagy from pro-survival to pro-death based on the threshold effect 50.

**Figure 2:**
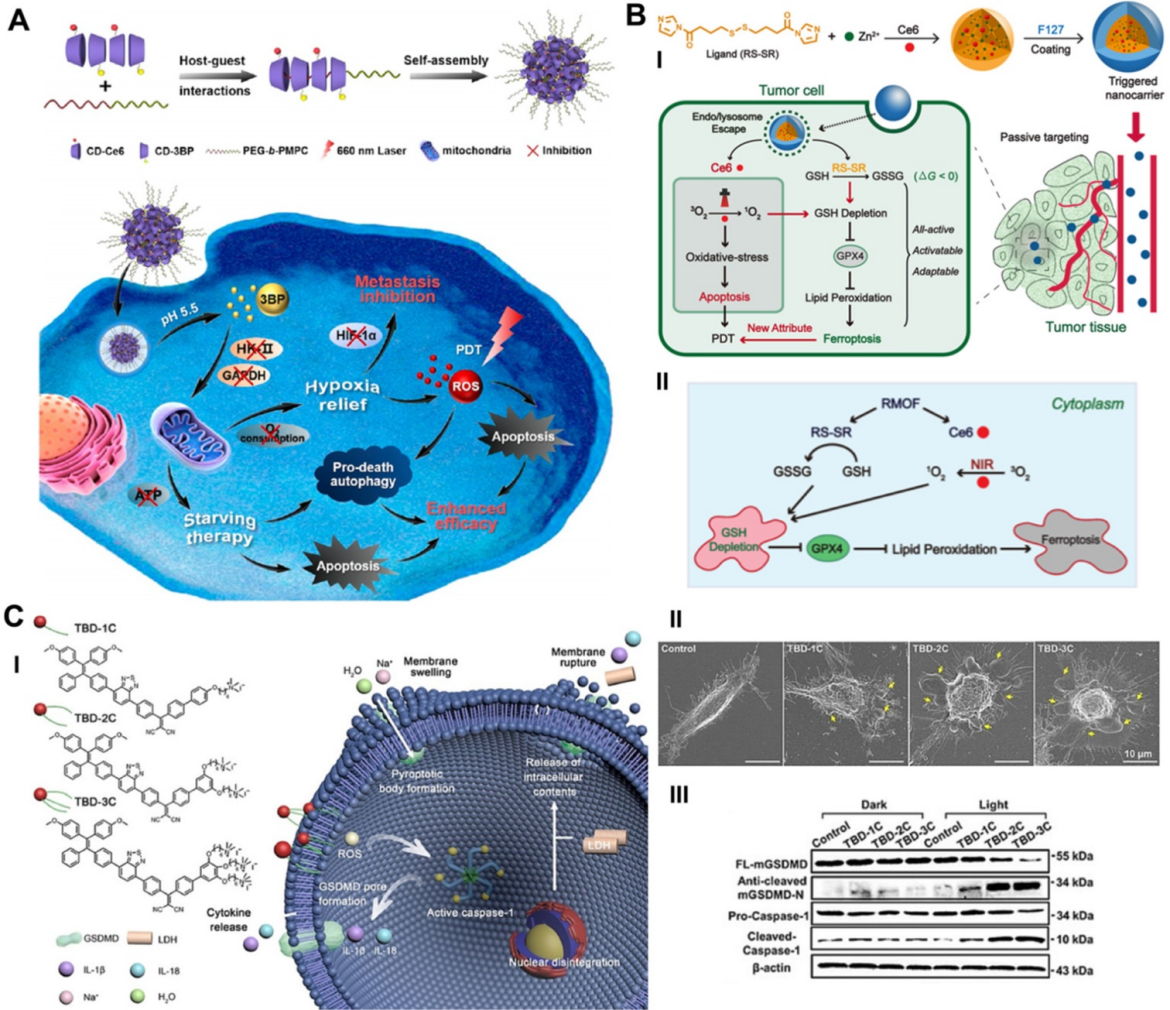
Schematic illustration of targeting RCD-augmented PDT, including **(A)** 3‐BP and Ce6 co-delivered CD-based nanoparticles induced pro-death autophagy for enhanced PDT. Adapted with permission from Ref. 51, copyright 2020 American Chemical Society. **(B)** The synthesis route of all-active photosensitive MOF and the anti-tumor mechanism of PDT by triggering apoptosis/ferroptosis. Adapted with permission from Ref. 53, copyright 2019 American Chemical Society. **(C)** Illustration of the pyroptosis pathway activated by three membrane anchoring AIE photosensitizers TBDs and representative scanning electron microscopy and WB results. Adapted with permission from Ref. 28, copyright 2020 WILEY-VCH Verlag GmbH & Co. KGaA, Weinheim.

Deng *et al.* constructed a supramolecular nanoplatform by integrating the α-cyclodextrin (CD)-based respiration inhibitor 3-bromopyruvate (3BP) and photosensitizer chlorin e6 (Ce6) with poly (ethylene glycol)-b-poly (2-methacryloyloxyethyl phosphorylcholine) (PEG-b-PMPC) via host-guest self-assembly interactions (CD-Ce6-3BP NPs), realizing excessively activated autophagy-strengthened photodynamic tumor suppression (**Figure 2A**) 51. On the one hand, 3BP reduced the consumption of O_2_ by restraining respiration, thereby alleviating tumor hypoxia and controlling tumor metastasis; on the other hand, 3BP-restrained respiration cut off the energy supply and resulted in starvation-induced autophagy. Excessive activation of autophagy by the consolidation of ROS production and starvation activation reversed the effect of cellular autophagy from promoting survival to promoting death, effectively augmenting the antiapoptotic efficacy of photodynamic tumor intervention. Compared with the 40.1% cell viability of CD-Ce6 NPs under laser irradiation, CD-Ce6-3BP NPs obtained an improved toxic effect of only 19%. The results of antitumor evaluation *in vivo* revealed that the combination therapy of 3BP and PDT not only thoroughly inhibited the growth of orthotropic tumors but also favorably suppressed metastasis to the lung. The apparently elevated and activated autophagy level plays a role in promoting tumor cell apoptosis, thus synergistically increasing the tumor treatment efficiency of PDT. Triggering pro-death autophagy provides an afflatus for future novel methodologies of cancer intervention therapy.

#### Targeted ferroptosis-assisted PDT

Presumably, ferroptosis targeting could be another distinct dedication to cancer cell death in the antitumor process of PDT. The primary indicator of ferroptosis is the iron-dependent accumulation of lipid peroxidation, which is generally maintained at a normal physiological state by the ferroptosis suppressor protein-1 (FSP1)-coenzyme Q10-NAD(P)H system and the GPX4-GSH-cysteine axis 15, 52. Disturbances in any part of the protective systems may lead to unconstrained lipid peroxidation, thus inducing ferroptotic cell death. Based on the above, Meng *et al.* constructed a Ce6-loaded metal organic framework (MOF) nanocarrier in which the disulfide-bearing imidazole ligand of MOF acted as the GSH scavenger to induce ferroptosis, and loaded Ce6 acted as a photosensitizer to trigger PDT (**Figure 2B**) 53. The all-active MOF was synthesized through the coordination of disulfide-containing imidazole organic ligands and metal zinc (Zn^2+^). The disulfide-thiol exchange reaction of MOF-induced GSH depletion enabled the loss of GPX4 activity and the induction of ferroptosis 54, significantly contributing to the antitumor effect by sensitizing PDT-induced cell apoptosis. The Ce6@MOF nanocarriers specifically accumulated in tumor sites observed in 4T1 breast tumor-bearing BALB/c nude mice by monitoring the intrinsic fluorescence of Ce6 after intravenous administration. After 20 days of intervention, the tumor volume and weight of the Ce6@MOF group showed the best antitumor efficacy compared to the other groups, which was consistent with the cytotoxicity results *in vitro*.

Along this line, Liu *et al.* also constructed a ferrous supply-regeneration nanonetwork to regulate tumor iron metabolism and achieve ferroptosis-enhanced PDT 55. A self-deposition network composed of Fe^3+^ (a ferroptosis inducer), tannic acid (TA, an acidity-activated reductant) and methylene blue (MB, a photodynamic agent) was constructed on sorafenib (SRF, a ferroptosis inducer) nanocrystals, resulting in nuclear corona SRF@Fe^III^TA-MB (SFT-MB) nanoengineering. In response to a lysosomal acidic environment, SRF@Fe^III^TA-MB nanoparticles can dissociate the corona and release SRF to inactivate the GPX4 enzyme for effective ferroptosis initiation, liberate TA to chemically reduce Fe^3+^ for iron redox cycling, emancipate MB to recover fluorescence, and perform photocatalytic reactions for imaging-guided PDT. SFT caused a lower level of GPX4 expression by western blot analysis and stronger green lipid peroxide (LPO) fluorescence by confocal laser scanning microscopy (CLSM) in 4T1 cells than in other groups, illustrating its ferroptosis activation ability. The Fe^2+^ supply in response to the acidic TME led to sustained cytotoxicity, which had a targeted killing effect on H_2_O_2_-overloaded cancer cells and less toxic side effects on normal cells. The inactivation of the GPX4 enzyme and the Fe^2+^-mediated Fenton reaction effectively led to the induction of ferroptosis, synergistically promoting MB-instructed photodynamic tumor therapy by increasing the sensitivity of cancer cells to apoptosis. The *in vivo* and *in vitro* results indicated that ferroptosis-induced and image-guided PDT based on SFT-MB achieved near to complete tumor elimination and held great promise for development as a powerful nanotherapeutic platform.

#### Activated pyroptosis-guided PDT

Due to its ability to release the contents of proinflammatory cells and trigger the immune response, pyroptosis has remarkable potential as an exceptional cancer cell death pathway, which may produce an auxiliary effect in photodynamic tumor ablation 56. Wu *et al.* reported the prospective capacity of membrane anchoring aggregation-induced emission (AIE) photosensitizers for photodynamic tumor therapy by activating the pyroptosis cell death pathway (**Figure 2C**) 28. By regulating the number of cationic chains connected to the aromatic structure, they synthesized three different AIE photosensitizers (TBD-1C, TBD-2C and TBD-3C). Under laser radiation, the generated ROS caused immediate membrane damage *in situ* and cytoplasmic lysis, subsequently resulting in efficient cancer cell pyroptosis/apoptosis removal. Furthermore, along with the increased number of cationic chains, the membrane anchoring ability of the photosensitizer was significantly enhanced, and the induced cell pyroptosis gradually occupied the dominant cell death pathway. Cell viability analysis demonstrated that the IC_50_ values of TBD-3C on 4T1 cells, HeLa cells and C6 cells were 7.1, 7.1 and 7.9 μM, respectively, under light irradiation, obviously less than those of TBD-1C (20.3, 24.0 and 33.4 μM) and TBD-2C (10.9, 11.7 and 13.4 μM), indicating the highest cancer cell killing efficiency of TBD-3C. The pyroptosis marker protein levels of GSDMD and caspase-1 and the concentrations of the proinflammatory cytokines IL-1β and IL-18 were both significantly improved in the TBD-R with light irradiation group, as measured by western blot and enzyme-linked immune sorbent assay (ELISA), respectively. Light-activated TBD successfully induced cell pyroptosis and triggered a series of antitumor immune responses, adding new aids to traditional photodynamic tumor therapeutics. This study exhibits a PDT-based pyroptosis-activated tumor intervention strategy and provides a new paradigm for broadening the application of RCD in PDT.

Targeting RCD in photodynamic tumor therapy exhibits a significant difference of molecular mechanism to traditional PDT-induced cell apoptosis, and the existence of multi-pathway cell death in PDT opened the novel avenues to resolve the difficult problems of tumor refractory, drug resistance, and heterogeneity from a more innovative angle. It also inspires us to explore other unknown therapeutic mechanisms of PDT and the synergistic antitumor effect between PDT and other RCDs, such as necroptosis, autophagy-dependent cell death and lysosome-dependent cell death. These emerging new cell death pathways not only enhance our understanding of tumors, but also provide ideas for the design of more efficient photodynamic tumor treatments.

### Sonodynamic Therapy

Compared with PDT, SDT triggered by ultrasound (US) has quickly grown to be a novel cancer therapeutic methodology owing to the advantages of deep tissue penetration, low cost and high convenience 57. US activates sonosensitizers to produce excessive high-energy oxygen-containing ROS via certain acoustic cavitation effects, such as sonoluminescence and pyroptosis, therein resulting in cancer cell apoptosis 58. However, the SDT-induced cell killing effect may be reversed by the inherent protective mechanism of cancer cells, resulting in weakened oxidative stress damage. It is highly expected to expand the cancer cell death-inducing ability of SDT by decreasing apoptosis resistance.

#### Blocking autophagy-improved SDT

**Figure 3:**
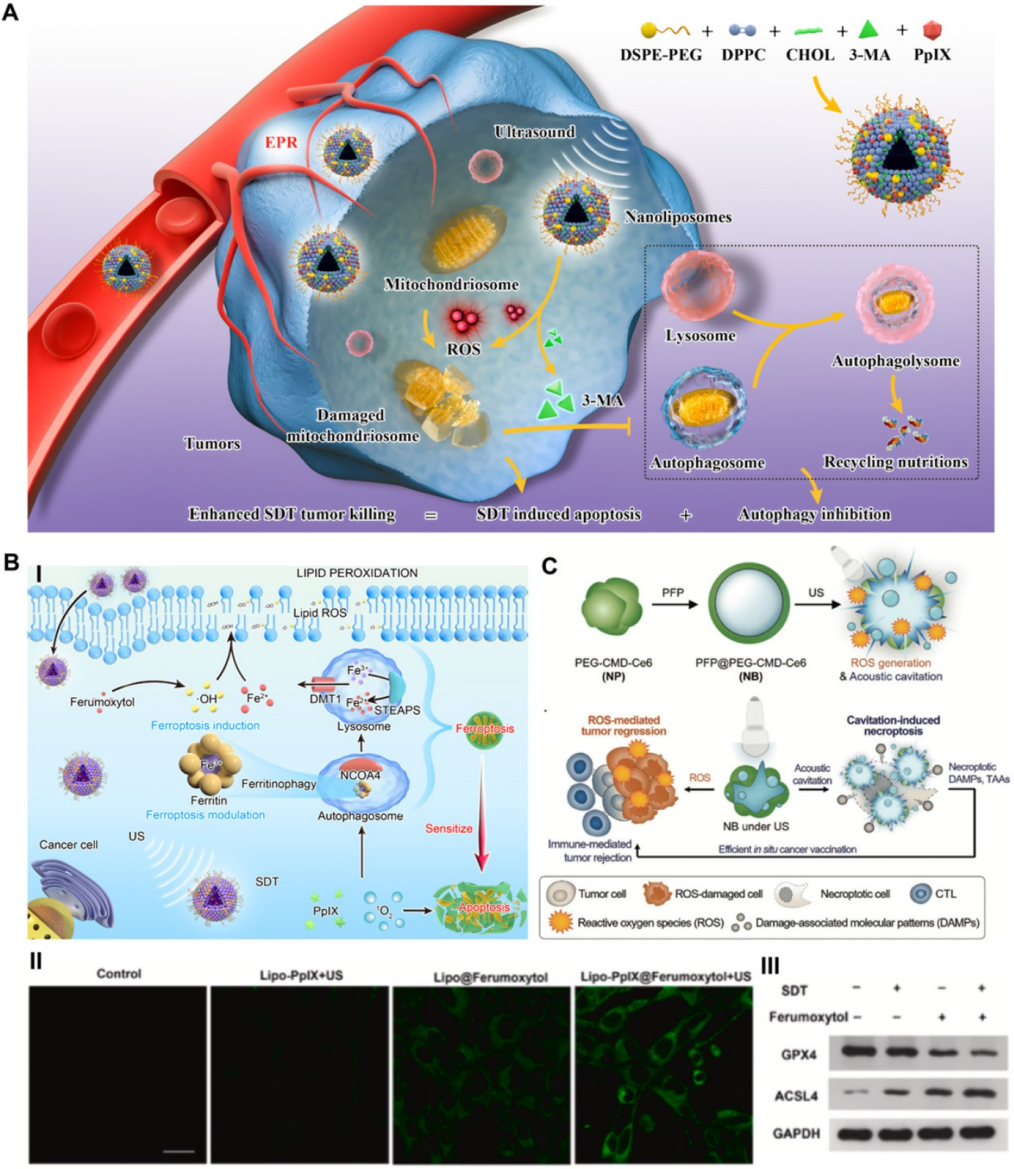
Schematic illustration of targeting RCD-enhanced SDT, including **(A)** the engineering PpIX/3-MA@Lip nanosonosentizer for synergistic SDT nanotherapeutics by autophagy blockage for fighting breast cancer. Adapted with permission from Ref. 59, copyright 2021 Springer Nature. **(B)** Schematic illustration of underlying cooperative tumor therapeutic mechanism of SDT-based ferroptosis-targeting and representative CLSM and WB results of ferroptosis. Scale bars, 30 µm. Adapted with permission from Ref. 61, copyright 2021 Elsevier. **(C)** Schematic illustration for the preparation of necroptosis-inducible NBs and their triggered anti-tumor immune response. Adapted with permission from Ref. 66, copyright 2020 WILEY-VCH Verlag GmbH & Co. KGaA, Weinheim.

Recently, our group reported an advanced collaborative tumor therapeutic strategy in view of autophagy inhibition-augmented SDT for treating ROS-resistant breast cancer 59. In detail, we proposed a comprehensive combination therapeutic strategy incorporating a sonosensitizer to enhance SDT and suppress autophagy, which was completed through the rational design of nanoliposome coloading of the early autophagy inhibitor 3-methyladenine (3-MA) and sonosensitizer protoporphyrin IX (PpIX) in a hydrophobic lipid bilayer by reverse evaporation (**Figure 3A**). The spherical nanoliposomes PpIX/3-MA@Lip, with an average hydrodynamic diameter of 143.2 nm and an average surface zeta potential of -35 mV, were synthesized by a typical reverse evaporation method. They exhibited high stability and long blood circulation time and effectively accumulated in tumor lesions through the typical enhanced penetration and retention (EPR) effect after tail vein injection, as shown by 24 h fluorescence imaging monitoring. SDT-induced cytoprotective autophagy analysis was evaluated by CLSM, flow cytometry, western blot and transmission electron microscopy (TEM). SDT caused the strongest DAPGreen fluorescence intensity at 0.5 h post US treatment, indicating successful autophagy, which was confirmed by increased LC3-II protein expression. After intervention with the autophagy inhibitor 3-MA, CLSM and TEM revealed a significant decrease in the number of autophagosomes caused by SDT intervention, revealing the effective inhibition of autophagy. The results of high-throughput mRNA sequencing showed that SDT prompted cellular self-protective autophagy by activating the MAPK signaling pathway and inhibiting the AMPK signaling pathway. The suppression of early autophagy by coloaded 3-MA notably reduced the resistance of cancer cells to ROS and achieved a significant additive effect on SDT by cutting off the circulating nutrient supply that meets the growth of cancer cells, achieving a 57.3% apoptosis rate *in vitro* via cellular flow cytometry analysis and a 89.32% tumor inhibition rate in an *in vivo* subcutaneous xenograft tumor model. The program of cooperatively combining SDT and autophagy blockade represents an effective paradigm for inhibiting refractory cancers.

Coincidentally, Qu *et al.* proposed a precise mitophagy inhibition strategy for promoting nanosensitizer-augmented sonodynamic glioma therapy 60. In detail, they designed an angiopep-2 peptide-modified nanoliposome coloading sonosensitizer Ce6 and late autophagy inhibitor hydroxychloroquine (HCQ) by a similar synthesis method (ACHL). The main difference is that Ce6 and HCQ are encapsulated in the hydrophobic lipid bilayer and hydrophilic core of nanoliposomes, respectively. Owing to the opening of the ultrasound-targeted microbubble destruction (UTMD)-mediated transient blood brain barrier (BBB), angiopep-2-modified nanoliposomes could selectively target glioma cells with high expression of low-density lipoprotein receptor-related protein 1 (LRP1) based on ligand-receptor binding. Under a second US stimulation, Ce6-generated ROS triggered cellular apoptosis and MAPK/p38-PINK1-PRKN-dependent mitophagy and simultaneously released HCQ blocked autophagosome degradation, thus augmenting the oxidative stress damage by SDT. Compared to the other groups of mice, the autophagy blockade/SDT group achieved longer survival and slower weight loss. Inhibition of mitochondrial autophagy increases the production of ROS in tumor cells and leads to oxidative damage and regeneration cycles, effectively increasing the efficiency of sonodynamic tumor therapy. The manipulation of mitophagy simultaneously with SDT-induced apoptosis offers novel insights into therapeutics for brain tumors.

#### Targeting ferroptosis-sensitized SDT

In addition to autophagy blockade-related improvements to SDT, we constructed a prospective nanoliposomal composite by encapsulating U.S. Food and Drug Administration (FDA)-approved pharmaceuticals ferumoxytol and PpIX based using the reverse evaporation method to develop an SDT-based oxidative ferrotherapy (**Figure 3B**) 61. The vintage encapsulation efficiencies of PpIX and ferumoxytol in nanoliposomes were up to 46.3 ± 2.2% and 34 ± 1.9%, respectively. In this novel nanosystem, the iron supplement ferumoxytol was applied as a ferroptosis inducer to import iron ions into cancer cells, which realized the metabolic reorganization of cancer cells and rendered tumors highly vulnerable to sonosensitizer-augmented SDT. Meanwhile, SDT also modulated critical ferroptosis checkpoints and improved ferroptosis sensitivity by promoting selective ferritinophagy, therefore implementing synergistic tumor suppression. Exploring the molecular mechanism by high-throughput transcriptome sequencing also verified that SDT-based ferrotherapy induced a dual signaling pathway involving apoptosis and ferroptosis. These Lipo-PpIX@Ferumoxytol nanoparticles, with all components approved by the US FDA, achieved a cooperatively improved antitumor effect on 4T1 breast cancer, with good safety, and exhibited remarkable clinical application potential. Quantitative assessment of early cell apoptosis showed that the combination therapy of SDT and ferumoxytol induced a total apoptotic ratio of 55.6%, which was significantly higher than those of the SDT group (8.9%) and ferumoxytol group (12.1%). The *in vivo* tumor suppression rate of the SDT/ferumoxytol group was 86.01%, while those of the simple SDT and ferumoxytol groups were only 51.5% and 55.54%, respectively. Considering that refractory tumors often demonstrate the drawbacks of insensitivity to SDT-induced apoptosis 62, the notion of dual ferroptosis/apoptosis RCD induction is a potential auxiliary strategy for inhibiting apoptosis-resistant tumors.

For the purpose of improving the SDT efficacy of hypoxic tumors, a manganese porphyrin-based metal-organic framework (Mn-MOF) nanoplatform was fabricated to self-boost O_2_ generation and reduce the intracellular GSH content to achieve ferroptosis-sensitized SDT 63. Mn-MOF was synthesized with biocompatible metal ion zirconium (Zr) as the joint combined with manganese 5,10,15,20-tetra (4-benzoic acid) porphyrin (Mn-TCPP) as the sonosensitive bridge ligand, which exhibited catalase-like activity to constantly generate O_2_ at the site of the hypoxic tumor by decomposing the H_2_O_2_ of the Mn element; GSH decreased the effect to induce ferroptosis of the Zr element, and the considerable ROS generation ability of TCPP under US irradiation was noted. The results of assays performed with an H_2_O_2_ detection kit showed that approximately 68.7% of H_2_O_2_ was decomposed by Mn-MOF in 8 h, which was higher than that of the Mn-TCPP group. Correspondingly, Mn-MOF increased O_2_ generation to 1.5 mg/L at a Mn concentration of 8 μg/mL. In addition, Mn-MOF significantly decreased the intracellular GSH content to 0.5 μM in H22 cells and to 1.0 μM in 4T1 cells after 24 h of treatment at a Zr concentration of 20 μg/mL. The IC_50_ values further confirmed the excellent killing effect of Mn-MOF for H22 cancer cells (10.4) and 4T1 cancer cells (14.25) under hypoxic conditions. Impressively, US-stimulated Mn-MOF not only induced dual apoptosis/ferroptosis but also enhanced the antitumor immunity response by improving the numbers of mature dendritic cells and activated CD8^+^ T cells and reducing the numbers of myeloid-derived suppressor cells (MDSCs) in H22 primary liver and 4T1 metastatic breast tumor models, thus showing its great potential as an advanced smart SDT nanosystem to achieve efficient suppression of hypoxic tumors.

#### Triggering necroptosis-enhanced SDT

As described, the most critical mechanism by which SDT inhibits tumors is ROS-induced oxidative stress damage 64. In addition, the cavitation effect of nanomaterials induced by US irradiation can promote the destruction of the plasma membrane and the release of biologically active damage-associated molecular patterns (DAMPs), thus facilitating the activation of the immune response 65. As a typical example, Um *et al.* reported a necroptosis-inducible polymeric nanobubble (NB) for heightened cancer sonoimmunotherapy (**Figure 3C**) 66. The innovative NBs were prepared by oil-in-water emulsion, using amphiphilic polymer conjugates as the carrier, and coloading with perfluoropentane (PFP) as the gas precursor and Ce6 as the sonosensitizer. US irradiation, on the one hand, activates Ce6 to induce ROS-mediated tumor regression. On the other hand, US triggers the vaporization of PFP, induces RIPK3/MLKL-independent necroptosis through acoustic cavitation, and ultimately promotes the release of biologically active DAMPs to achieve immune-mediated tumor rejection. The cytotoxic effects on CT26 cells showed that over 50% of cells were inhibited by SDT-generated ROS and acoustic cavitation-induced necroptosis. As a marker of DC maturation, the expression of CD86 in bone marrow-derived dendritic cells (BMDCs) stimulated with US and NB increased by 20% compared to that in the control group, as shown by flow cytometry analysis, indicating the strong immunogenicity of DAMPs released from necroptotic cancer cells. After combining immune checkpoint blockade therapy, NB/US treatment achieved 97.03% tumor inhibition in the CT 26 BALB/c mouse model during the 22-day monitoring period. The antitumor immune response induced by necroptosis and the tumor cell apoptosis induced by SDT complementarily inhibit the growth of *in situ* and metastatic tumors. This promising strategy shows that necroptosis induced by exogenous US stimulation can effectively enhance the antitumor response of SDT.

Although SDT has several advantages in tumor therapy, considering that refractory tumor cells are often insensitive to apoptosis, exploring other types of RCD has important sensitization significance. SDT mediated by autophagy inhibition, ferroptosis and necroptosis induction has gained considerable credit due to its huge therapeutic potential, and it also provides new ideas for broadening other nanomedicine applications.

### Photothermal Therapy

Noninvasive PTT induces tumor ablation by transforming light energy into hyperthermia with the help of photothermal agents and demonstrates remarkable clinical application potential 67. Several kinds of nanoparticles have been extensively investigated as near-infrared (NIR)-assisted photothermal agents and have shown excellent photothermal effects and superb biocompatibility, such as gold nanostructures 68, graphene 69 and other carbon nanomaterials 70. However, the heterogeneous distribution of PTT hyperthermia frequently leads to incomplete tumor eradication. Certain different combinations of PTT and other therapies, such as PDT, radiotherapy, and immunotherapy, have made great progress in enhancing the efficiency of tumor elimination 71-73. Therefore, it is logical and reasonable to explore the synergistic effect of the combination of RCD and PTT on inhibiting tumor progression.

#### Autophagy blockade-incorporated PTT

**Figure 4:**
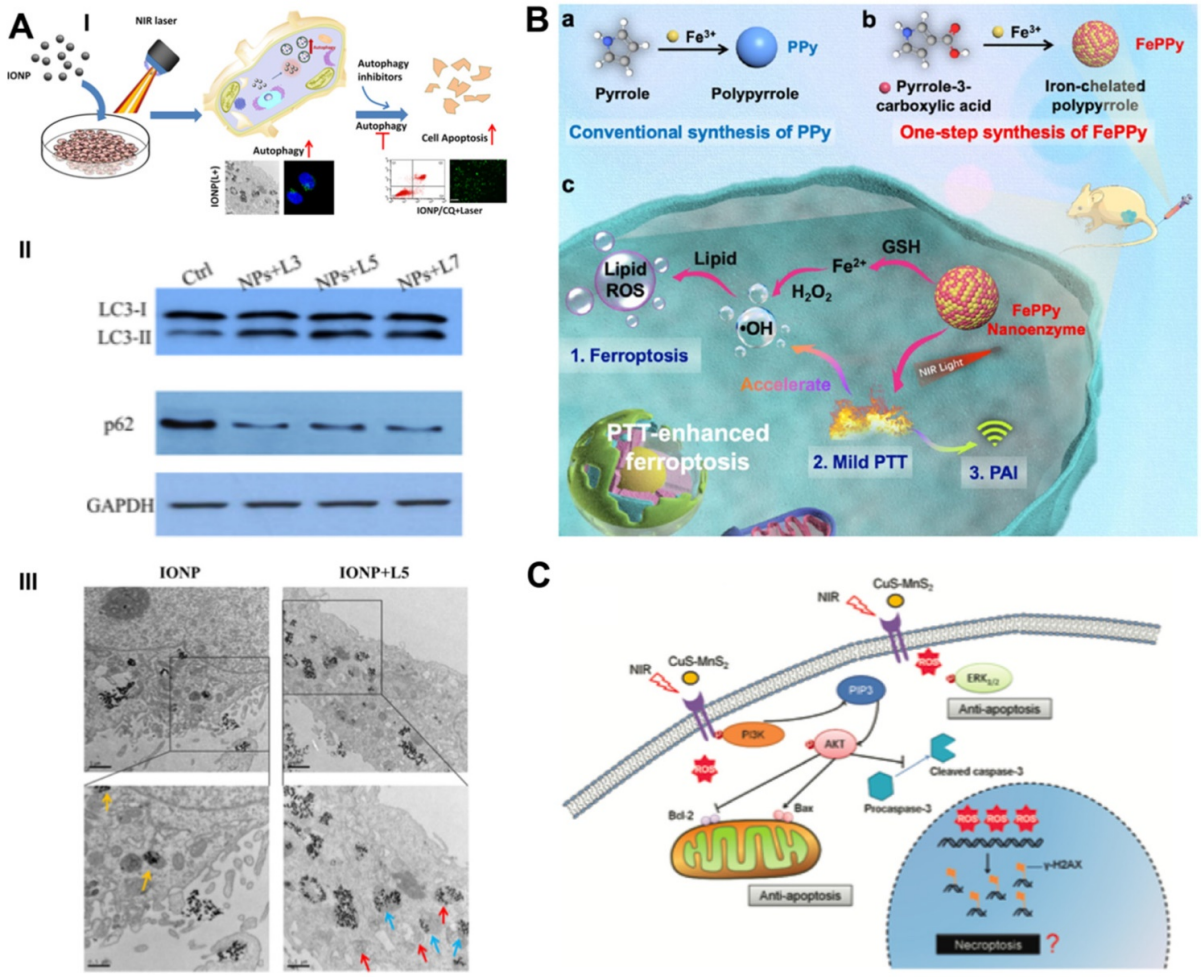
Schematic illustration of combinational cancer therapeutics by PTT induced dual RCDs. **(A)** Schematic illustration and representative mechanism results of photothermal effect induced cell self-protective autophagy by iron oxide nanoparticles and autophagy inhibition-enhanced cytotoxicity. Scale bars, 0.5 µm. Adapted with permission from Ref. 76, copyright 2018 American Chemical Society. **(B)** The synthesis routes of iron self-boosting polymer nanoenzymes, and the anti-tumor mechanism of PAI-guided PTT-enhanced ferroptosis. Adapted with permission from Ref. 80, copyright 2021 American Chemical Society, **(C)** Schematic illustration of CuS-NiS_2_ nanomaterials for photothermal gastric carcinoma therapy via triggering dual apoptosis and necroptosis. Adapted with permission from Ref. 82, copyright 2019 Royal Society of Chemistry.

The thermal effect of PTT leads to the destruction of cell contents, thereby activating cytoprotective autophagy. Autophagy-related tolerance establishes the resistance of cancer cells to various therapies and provides a possibility for autophagy suppression strategies to augment the efficacy of PTT. Zhou *et al.* developed chloroquine (CQ)-loaded polydopamine nanoparticles (PDAs) to assess the collaborative effect of autophagy blockade on PTT efficacy 74. The autophagy inhibitor CQ was loaded on the surface of PDA via interactions with π-π stacking 75^,^ and the loading efficiency was determined by the linear calibration curve between the concentration of CQ and the homologous peak intensity at a 344-nm wavelength. PDA/CQ nanoparticles selectively released CQ under stimulation of the moderately acidic tumor microenvironment (TME) at a pH of 6.0, thereby effectively inhibiting the activity of autophagolysosomes. The cell viability of CQ/PTT-treated HeLa cells was largely reduced by approximately 80%, while the PTT group achieved only moderate suppression - approximately 40%. Two doses of NIR irradiation and three injections of nanomaterials were administered during the 9-day *in vivo* intervention period. Under a laser power density of 3.0 W/cm^2^, PDA-PEG/CQ almost completely stopped tumor growth in MDA-MB-231-bearing mice at a low tumor-site temperature of approximately 42 °C, which was significantly more advantageous than PTT alone. Systematic experimental studies have indicated that autophagy blockade remarkably heightens the efficacy of mild PTT by blocking the recycling of nutrients, resulting in effective tumor suppression. The same phenomenon was also observed in MCF7 breast cancer cells receiving CQ-enhanced photothermal treatment with iron oxide nanoparticles (IONPs) (**Figure 4A**) 76.

PTT-activated autophagy is also intently related to the shape and composition of photothermal agents. Zhang* et al.* synthesized three types of copper (Cu)-palladium (Pd) alloy photothermal agents, including two tetrapod nanoparticles with similar sizes and shapes but different Cu contents (42% for TNP-1 and 13% for TNP-2) and one spherical nanoparticle with the same composition (SNP) 77. The temperature elevation profiles showed that the temperature of the SNP, TNP-1 and TNP-2 dispersions improved to 45 °C, 67 °C and 71 °C after 5 min of irradiation. The results of ultraviolet-visible-NIR spectra also demonstrated that TNPs obtained better photothermal conversion efficiency (PCE) than SNPs due to the special sharp-tip structure, and TNP-2 exhibited superior absorption than TNP-1 owing to its higher Pd ratio 78. By evaluating the expression of LC3 protein in HeLa cervical cancer cells, they found that only the TNP-1 nanophotothermal agent induced complete cellular self-protective autophagy in a way that depended on the shape and composition. NIR irradiation plus 3-MA remarkably improved the cytotoxicity of TNP-1 and caused HeLa cell viability to decrease by 88%, which was notably higher than TNP-1-mediated PTT alone. After combinational intervention with the autophagy inhibitor 3-MA or CQ in 4T1 and MCF7 breast cancer models, TNP-1 achieved a remarkably enhanced tumor inhibition effect under 808-nm NIR irradiation, with a 92% reduction in tumor weight. These results provide a paradigm for an optimized PTT scheme by inhibiting prosurvival autophagy, which is especially beneficial for eliminating drug-resistant tumors.

#### Ferroptosis regulation-cooperated PTT

To increase the sensitivity of PTT-mediated cancer cell death, Ou *et al.* constructed cysteine-depleted organic charge transfer (CT) photothermal agents by supramolecular assembly of 3,3',5,5'-Tetramethylbenzidine (TMB) with the multicyano substituted acceptor 2,3,5,6-tetrafluoro-7,7,8,8- tetracyanoquinodimethane (F4TCNQ) 79. TMB-F4TCNQ complex nanoparticles presented a high CT degree, strong NIR-II absorption ability and ideal photothermal effect owing to the intermolecular CT from TMB to the F4TCNQ acceptor. Specifically, the CT complex nanoparticles can be effectively degraded due to the selective reaction between cysteine and arylnitriles of F4TCNQ. The depletion and lack of cysteine directly results in the inhibitory effect of GSH biosynthesis and intracellular redox imbalance, thus contributing to the triggering of ferroptosis. TMB-F4TCNQ exhibited low cytotoxicity and outstanding biocompatibility, as 4T1 and HeLa cells maintained a high viability of more than 80% at an incubation concentration of 60 μg/mL. Under 1060 nm laser irradiation of 1.0 W/cm^2^ for 5 min, the cell viability of 4T1 and HeLa cells gradually decreased as the incubation concentration of TMB-F4TCNQ increased. The *in vivo* therapeutic efficacy was further evaluated in a 4T1 tumor-bearing mouse model, and an inhibition rate of 43% was achieved, showing high tumor suppression efficiency. In addition, TMB-F4TCNQ can be utilized to perform photoacoustic imaging (PAI) to instruct exact photothermal tumor therapy *in vivo*. The combination of NIR-II-activated PTT and GSH consumption-instructed ferroptosis based on cysteine-responsive CT complexes resulted in outstanding tumor killing efficiency and demonstrated a prospective paradigm in tumor ferrotherapy and phototheranostics.

In addition to the regulation of the GPX4-GSH-cysteine axis to trigger ferroptosis, Cui *et al.* proposed an iron self-boosting strategy for enhanced PTT/ferrotherapy based on rationally designed polymer nanoenzymes (**Figure 4B**) 80. By applying iron as an oxidant and pyrrole-3-carboxylic acid as a monomer, they synthesized an iron self-boosting polymer FePPy nanoenzyme through a simple one-step method. Iron ions can be substantially reserved in FePPy nanoparticles due to their strong chelating ability to pyrrole-3-carboxylic acid and are supplemented by self-boosting to comply with Fenton reaction-based ferroptosis. The highly active reaction between Fe^3+^ and S oligopeptides in GSH resulted in effective GSH consumption, thus enhancing the oxidative ability of the Fenton reaction. The GSH consumption rate of FePPy reached 34.7%, as measured by the absorbance of 5,5′-dithiobis-(2-nitrobenzoic acid) (DTNB), and laser irradiation significantly improved it to 57.3% due to the thermal acceleration effect. Because of the presence of iron, FePPy nanoparticles exhibit satisfactory low-temperature photothermal effects. Correspondingly, the photothermal effects enable the acceleration of the iron ion-instructed Fenton reaction, thus achieving synergistically heightened photothermal apoptosis and ferroptosis. Upon 808 nm laser irradiation at 0.6 W/cm^2^ for 5 min, the HeLa cell viability of the FePPy group decreased to less than 20%, while that of only the PPy group reached over 40%. The *in vivo* results demonstrated that FePPy induced almost complete tumor elimination under light irradiation, which is fairly superior to the suppression efficacy of PPy, indicating the cooperative enhanced therapeutic effect of ferroptosis on PTT.

#### Necroptosis induction-augmented PTT

Chen *et al.* designed a novel ternary copper-based chalcogenide nanomaterial, CuS-NiS_2,_ for combinational phototherapy of gastric carcinoma by triggering Bcl-2/Bax pathway-induced cell apoptosis and MLKL/CAPG pathway-induced cell necroptosis under 808 nm NIR irradiation 81. The flower-like structure of CuS-NiS_2_ nanoparticles was synthesized by hydrothermal and subsequent calcination with diameters of approximately 300-500 nm. Under laser irradiation, the CuS-NiS_2_ nanoplatform not only demonstrated an exceptional PCE of 52.2% but also enhanced T1- and T2-weighted MRI signals, thus implementing MRI-guided PTT-induced tumor necroptosis/apoptosis therapy. Western blotting analysis showed that PTT reduced the protein expression of Bcl-2 and increased the protein expression of Bax and phosphorylated MLKL, thus providing strong evidence of the anticancer mechanisms of CuS-NiS_2_ nanoparticles under NIR irradiation. The CuS-NiS_2_ nanomaterials resulted in 62.1% and 69.3% cell death rates in AGS and MKN-45 cells, respectively, under 808-nm NIR laser irradiation for 5 min. *In vivo* assessment demonstrated that the growth rate of gastric tumors was greatly reduced by PTT-induced dual apoptosis/necroptosis during the 15-day monitoring period. Another CuS-MnS_2_ nanoflower with a similar chemical composition also exhibited a PTT necroptosis-inducing effect on ovarian cancer (**Figure 4C**) 82.

The RCD regulation of autophagy/ferroptosis/necroptosis obviously increases the cytotoxicity of PTT by new regulated tumor cell death-sensitized apoptosis induction. The anticancer mechanism of RCD induced by photothermal agents under NIR irradiation provides a new paradigm for broadening the application scope of photothermal tumor therapy.

### Immunotherapy

Compared with cytotoxic radiochemotherapy or molecular targeted therapy, cancer immunotherapy is altering the field of oncology owing to its superior therapeutic outcomes philosophically and practically 83. Generally, immunotherapy mainly depends on the activation of the T-cell immune response by boosting the infiltration of cytotoxic T lymphocytes (CTLs) into tumors and demonstrates great potential to eradicate established tumors and prevent tumor recurrence or metastasis 84. Nevertheless, the proportion of patients who respond to effective immunotherapy remains modest, with an objective response rate of only approximately 15%, as tumors have multiple means of immune evasion, such as displaying relatively low immunogenicity to escape the recognition of immune cells. Breaking local immune tolerance at the tumor site to trigger an antitumor immune response is still a daunting challenge. The latest developments in the field of nanotechnology and bioengineering can significantly improve the safety and efficacy of cancer immunotherapy, bringing new hope that the therapeutic index can be maximized 85.

#### Mediating autophagy-amplified immunotherapy

**Figure 5:**
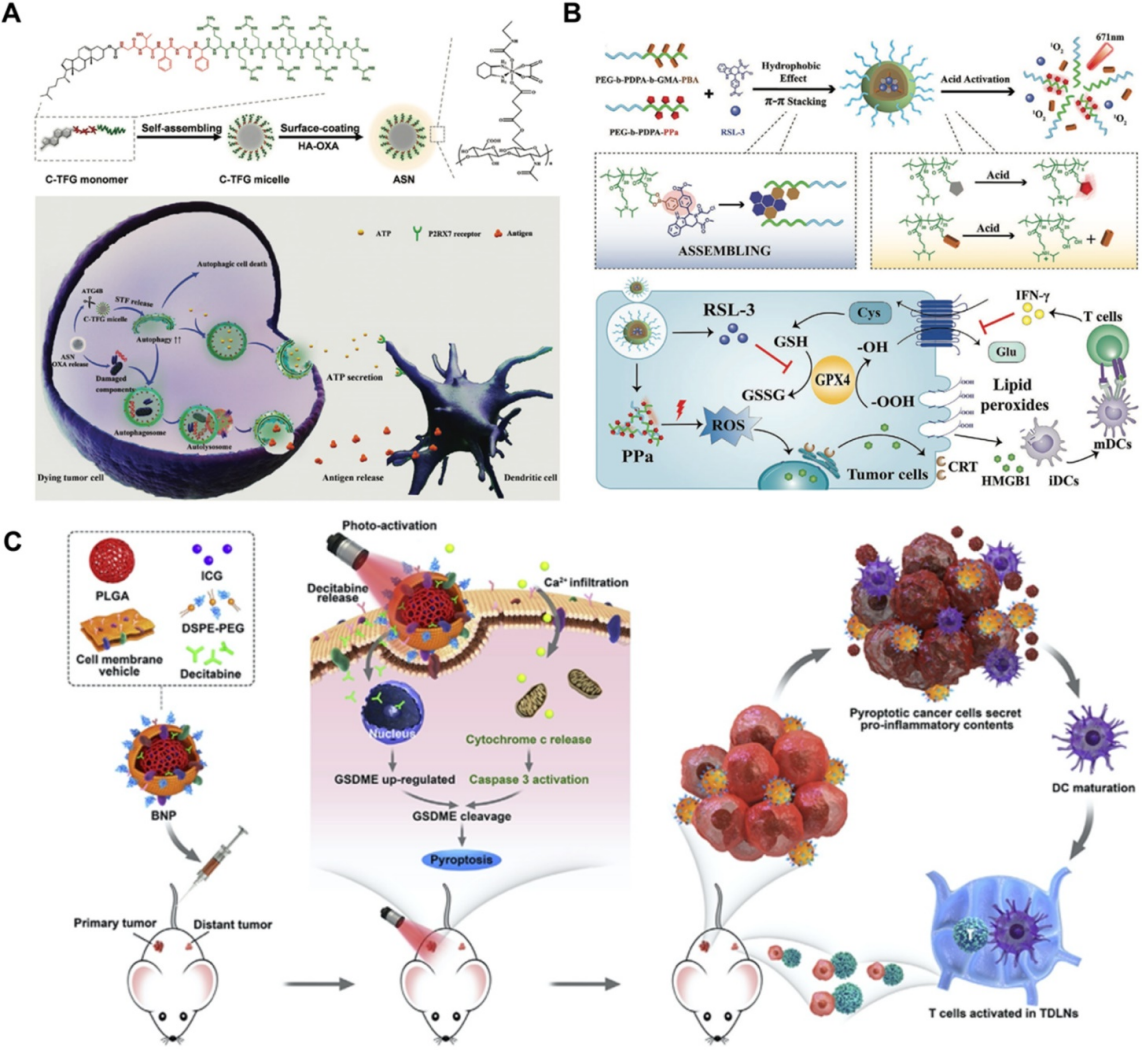
Schematic illustration of RCD-augmented immunotherapy, including **(A)** the main synthesis procedure of autophagy responsive nanoparticles ASN and anti-tumor mechanism of OXA-mediated immunogenic cell death and autophagy-promoted DCs recruitment. Adapted with permission from Ref. 91, copyright 2020 WILEY-VCH Verlag GmbH & Co. KGaA, Weinheim. **(B)** Schematic illustration of acidity-activatable dynamic nanoparticles for improving cancer immunotherapy by boosting ferroptotic cancer cell death. Adapted with permission from Ref. 95, copyright 2021 WILEY-VCH Verlag GmbH & Co. KGaA, Weinheim. **(C)** The mechanism of photo-activated cancer cell pyroptosis for enhanced solid tumor immunotherapy. Adapted with permission from Ref. 30, copyright 2020 Elsevier.

Chemotherapeutics can potently mediate the antitumor immune response by releasing DAMPs, inducing immunogenic cancer cell death, and stimulating intratumor T-cell infiltration 86. In addition, autophagy induction plays a vital role in antigen presentation and immune cell recruitment 87. As a double-edged sword, different levels of autophagy usually result in diametrically opposite cell fates. Unfortunately, chemotherapy usually induces “mild autophagy”, which plays a protective role in resisting drug treatment by eliminating harmful intracellular contents and supplementing beneficial substrates and energy to repair damaged DNA 7, 88. Only excessively activated autophagy can lead to autophagic cell death, which increases chemosensitivity and immune activity 89, 90. To overcome this problem, Wang *et al.* designed an autophagy-responsive drug release nanosystem to achieve on-demand autophagy cascade amplification and boost cancer immunotherapy (**Figure 5A**) 91. Autophagy-sensitive nanoparticles (ASNs) were prepared by self-assembly of C-TFG micelles and electrostatic binding of the chemotherapeutic prodrug hyaluronic acid oxaliplatin (HA-OXA). C-TFG micelles serve as the core of ASN and simultaneously load the autophagy inducer STF-62247. After reaching the tumor site, HA-OXA is degraded to release OXA under stimulation of the TME, triggering mild autophagy. The autophagy-related gene 4 (ATG4) enzyme cleaves TFG peptides to release the autophagy inducer STF-62247 from C-TFG micelles, leading to an overactivated state of autophagy, thereby promoting antigen presentation of dying cells. Flow cytometry analysis showed that ASN not only led to the highest recruitment ratio of immature DCs of up to 28.1 ± 1.5% but also stimulated 1.3-fold DC maturation and 1.4-fold Th1 lymphocyte (CD3^+^CD4^+^IFN-γ^+^) and CD8^+^ T cell (CD3^+^CD8^+^IFN-γ^+^) recruitment compared with the OXA/STF group. In summary, intelligently activated autophagy significantly improved the antitumor efficiency of immunotherapy by promoting tumor antigen presentation and immune cell recruitment. Its antitumor efficacy was also observed in CT26-bearing BALB/C mice; the ASN group achieved the slowest tumor growth rate, which was 9.1-fold and 2.27-fold lower than that of the OXA group and AIN group, respectively.

#### Boosting ferroptosis-promoted immunotherapy

Limited by the insufficient immunogenicity of tumor cells, cancer immunotherapy shows a relatively low response rate and therapeutic index. Fortunately, the proinflammatory cytokine interferon γ (IFN-γ) secreted by tumor-infiltrating T lymphocytes can induce ferroptotic cell death by inhibiting the expression of endogenous SLC7A11 and SLC3A2 proteins, and the formed LPO acts as a localization signal to promote the phagocytosis of tumor cells by dendritic cells (DCs), thereby eliciting the immunogenicity of tumor cells 92, 93. On this basis, targeted delivery of GPX4 inhibitors such as RSL-3 to tumors can further significantly amplify ferroptosis-mediated immunotherapy 94. Consistent with this line of thinking, Song *et al.* designed an acidity-activated dynamic nanoplatform for targeted delivery of RSL-3 and PDT to effectively promote cancer immunotherapy (**Figure 5B**) 95. BNP@R nanoparticles were synthesized by integrating an ionizable block copolymer and acid-liable phenylboronate ester (PBE) dynamic covalent bonds to package RSL-3 into a hydrophobic core through the interaction of π-π stacking and conjugate with the photosensitizer pheophorbide a (PPa). After being engulfed by cell lysosomes, the nanoparticles were activated by acid-triggered dynamic covalent cleavage of PBE and protonation of the hydrophobic core to release PPa and RSL-3, realizing PDT-sensitized ferroptosis-enhanced immunotherapy. However, BNP@R-induced PDT and ferroptosis significantly triggered programmed death ligand 1 (PD-L1) upregulation owing to intratumoral T lymphocyte infiltration and IFN-γ secretion, thus laying the foundation for PD-L1 blockade. Combined with αPD-L1, the BNP@R nanoplatform under laser irradiation effectively caused more than 2.0-fold higher intratumoral infiltration of IFN-γ^+^CD8^+^ T lymphocytes, dramatically inhibited the growth of B16-F10 melanoma tumors and the metastasis of 4T1 breast tumors, and significantly improved the survival rate of tumor-bearing mice by 35% within 50 days of the final treatment, indicating the prospective potential of ferroptosis-promoted immunotherapy.

#### Triggering pyroptosis-raised immunotherapy

The therapeutic effect of immunotherapy on solid tumors suffers from a downregulated or suppressed systemic immune response 96. As a kind of inflammatory immunogenic cell death, pyroptosis presents a new strategy for inhibiting solid tumors by relieving immunosuppression and promoting the systemic immune response 97. Based on this, Zhao *et al.* designed biomimetic nanoparticles (BNPs) to achieve photoactivated and pyroptosis-raised immunotherapy for solid tumors by fusing breast cancer membranes onto a poly (lactic-co-glycolic acid) polymeric core and co-encapsulating indocyanine green (ICG) and decitabine (DCT) (**Figure 5C**) 30. Benefiting from the tumor-homing properties of cancer cell membranes, BNP effectively targeted and accumulated in solid tumors. Subsequently, the ICG-mediated photoactivation effect apparently improved the intracellular Ca^2+^ concentration, thereby promoting the release of cytoplasmic cytochrome c and the activation of caspase-3. DCT molecules synergistically facilitated the expression of GSDME through DNA methylation, which contributed to caspase-3 cleavage and induced stronger cancer pyroptosis. Activated inflammatory cell pyroptosis effectively promoted the maturation of DCs and induced antitumor immunity, thus exhibiting a strong suppressive effect on the growth and distant metastasis of primary breast tumors. The *in vivo* tumor inhibition effect was evaluated by developing a bilateral 4T1 tumor-bearing mouse model. After a 28-day therapeutic period, 67% of the primary tumors and 33% of distant tumors were eliminated by treatment with BNP plus photoactivation, and a 60-day survival cycle of 100% mice was achieved in this group.

Due to the weak immune response, the effect of tumor immunotherapy is far from the expected goal. The combination of RCD and immunotherapy represents the current advanced tumor treatment strategy, especially the use of nanomedicine to achieve the regulation of autophagy, ferroptosis or pyroptosis, which can more fully elicit immunogenicity and more efficiently stimulate immune response, thereby improving the inhibitory effect of immunotherapy on tumor cells.

### Chemodynamic Therapy

Nanocatalytic medicine has been recently developed as a promising tumor intervention by triggering actual redox reactions for excessive production of highly toxic ROS based on Fenton or Fenton-like reactions 98. Driven by the latest advances in nanochemistry, a large number of nanocatalysts have been utilized to tumor sites to launch catalytic reactions and regulate the TME to induce therapeutic effects. Based on the different biological metabolic pathways of cancer cells and normal cells, CDT applies exclusive biochemical states, such as mild acidity and excessive H_2_O_2,_ to promote special chemical reactions, especially the Fenton reaction in tumors. It is believed that this catalytic treatment modality will play an increasingly significant role in tumor nanomedicine.

#### Autophagy inhibition-combined CDT

**Figure 6:**
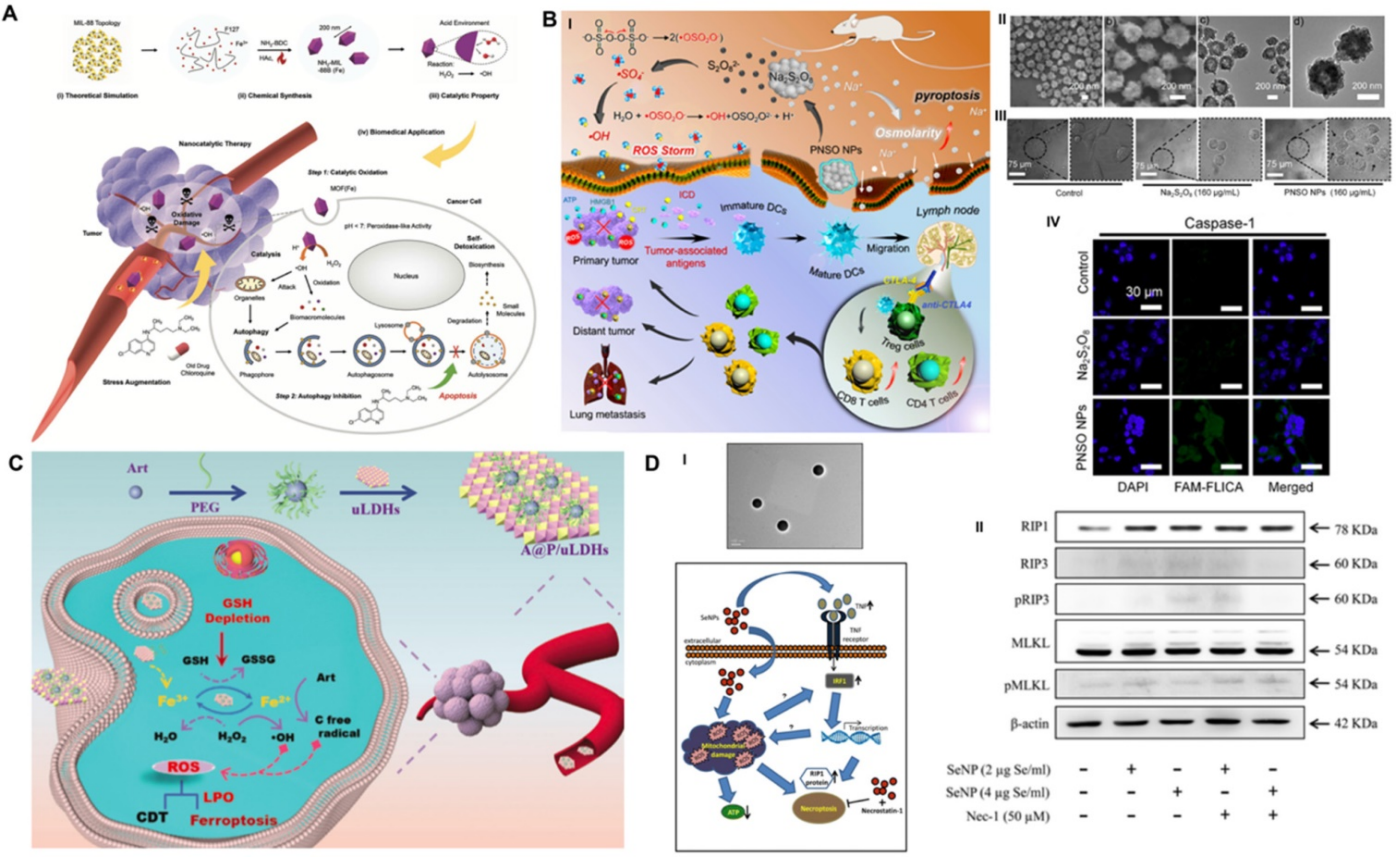
Schematic illustration of RCD manipulation-heightened CDT. **(A)** The synthesis route of MIL-88 MOF nanoparticles and their main anti-tumor mechanism of autophagy inhibition assisted Fenton catalytic reaction. Adapted with permission from Ref. 99, copyright 2020 WILEY-VCH Verlag GmbH & Co. KGaA, Weinheim. **(B)** The mechanism of Na_2_S_2_O_8_ nanoparticles triggering pyroptosis to improve tumor immunotherapy through ROS storm and elevated tumor osmotic pressure and representative electron microscopy and CLSM results. Adapted with permission from Ref. 102, copyright 2020 American Chemical Society. **(C)** Schematic diagram of the farbrication of A@P/uLDHs nanohybrids, and the proposal therapeutic mechanisms of A@P/uLDHs for ferroptosis-assisted CDT. Adapted with permission from Ref. 103, copyright 2021 WILEY-VCH Verlag GmbH & Co. KGaA, Weinheim. **(D)** TEM image of selenium nanoparticles extracted and purified from B. licheniformis, as well as the proposed mechanism of selenium induced necroptosis in PC-3 cells. Adapted with permission from Ref. 40, copyright 2017 Springer Nature.

CDT-activated redox chemical reactions promote the conversion of intracellular H_2_O_2_ to •OH and cause effective oxidative damage in cancer cells but suffer from discounted suppression efficiency owing to the prosurvival mechanism of autophagy 59. To overcome this obstacle, Yang *et al.* implemented a pharmacological strategy of autophagy inhibition to heighten ROS-induced oxidative damage based on the late autophagy inhibitor CQ-combined Fe-MOF nanocatalyst (**Figure 6A**) 99. Nanoscale MOF NH_2_-MIL-88B (Fe) was synthesized with Fe(III) salt and NH_2_-BDC through the hydrothermal route 100. It demonstrated intrinsic peroxidase-like activity to disproportionate cellular H_2_O_2_ into highly toxic •OH under the stimulation of the TME, which seriously damaged proteins and organelles of cancer cells and activated prosurvival autophagy. Moreover, appropriate interventions for the process of autophagic degradation by CQ effectively cut off the self-protection pathway and induced an amplified toxic therapeutic effect of Fe-MOF. As the concentration of MOF and CQ increased, the viability of A375 and HeLa cancer cells was substantially decreased by the synergetic treatment, which displayed a “1 + 1 > 2” additive effect compared with these monotherapies. It is noted that synergistic CDT and autophagy inhibition therapy resulted in a highly more evident antineoplastic effect than the linear addition of two single monotherapies, as demonstrated by 77.97% vs. 50.02% efficacy for the A375 model and 73.56% vs. 53.87% efficacy for the HeLa model. Comprehensive research data suggested that this combined treatment method obtained extraordinary antineoplastic effects on both malignant melanoma (A357) and cervical cancer (HeLa), which may have guiding significance for the design of future treatment options.

#### Pyroptosis initiation-collaborated CDT

The relatively low O_2_ and H_2_O_2_ content in the TME and strict pH conditions of the Fenton reaction limit the ROS production capacity of CDT and restrict the therapeutic effect of ROS-guided dynamic tumor therapy 101. To improve the CDT-mediated tumor inhibition effect, Liu *et al.* first synthesized phospholipid-modified sodium persulfate (Na_2_S_2_O_8_) (PNSO) nanoparticles through the reaction of sodium oleate with ammonium persulfate for in site gradual degradation to Na^+^ and S_2_O_8_^2-^ and changing to toxic ROS (^•^SO_4_^-^ and ^•^OH), which was not affected by the amount of H_2_O_2_ in or pH value of the TME (**Figure 6B**) 102. In addition, PNSO nanoparticles bypassed the cell ion transport rules and transported a great amount of Na^+^ into cancer cells via endocytosis, leading to a surge in cell osmotic pressure and rapid cell lysis, which initiated unusual caspase-1-associated pyroptosis. More importantly, the high immunogenic cell death induced by synergistic PNSO nanoparticle treatment can regulate the immunosuppressed TME and trigger antitumor immune responses, thereby eradicating tumor metastasis and recurrence. Ultimately, the synergistic effect of ROS and osmotic pressure-induced pyroptosis effectively eliminated tumors. The results of ELISA demonstrated that the secretion of cytokines associated with DC maturation, such as interleukin 6 (IL-6), tumor necrosis factor α (TNF-α), and interleukin 12 (IL-12p70), was significantly increased, and the immunosuppressive cytokine interleukin 10 (IL-10) was decreased, indicating the promaturation role of PNSO nanoparticles for DCs. To enhance the anti-neoplastic effect *in vivo*, anti-cytotoxic T-lymphocyte-associated protein 4 (anti-CTLA4) was selected for combination with PNSO nanoparticles to block CTLA4 and activate T cell immune function, and both primary and distant metastasis tumors were almost completely eliminated by this intervention. This work has broadened the horizon for the application of RCD in CDT antitumor nanomedicine.

#### Ferroptosis induction-assisted CDT

As a novel RCD form, ferroptosis induction provides an alternative solution to improve the antitumor efficacy of traditional CDT-induced apoptotic cell death. The ferron iron-triggered Fenton reaction used for ROS production has been proven to be a valid methodology to target ferroptosis. In addition to delivering excessive iron-based nanoparticles into the cell, it is necessary to ensure that there is enough H_2_O_2_ in the cell to perform the Fenton reaction. Based on this idea, Yang* et al.* fabricated an innovative self-assembled nanohybrid by loading PEG-encapsulated artemisinin (Art) on ultrathin MgFe-layered double hydroxide (uLDH) nanosheets (represented as A@P/uLDHs) to overcome the defect of cancer cell apoptosis resistance and achieve ferroptosis-enhanced CDT (**Figure 6C**) 103. The Fe^3+^ released from transitional metal-based LDH nanosheets could be responsively reduced to Fe^2+^ by GSH in the TME, which then activated loaded Art, an endoperoxide-containing sesquiterpene isolated from the Chinese herbal medicine Artemisia annua, to produce cytotoxic C-centered free radicals independent of endogenous H_2_O_2_ for continuous CDT. In addition, the self-cycling generation of ROS and the consumption of GSH could further oxidize unsaturated fatty acids to produce LPO, which could induce ferroptosis and sensitize CDT-induced cell apoptosis. CLSM observation of intracellular ROS with DCFH-DA indicated that HeLa cells incubated with A@P/uLDHs exhibited the strongest green fluorescence under simulated TME conditions. In addition, Ferro-Orange and DTNB (Ellman's reagent) assays suggested that A@P/uLDHs effectively elevated Fe^2+^ accumulation and GSH consumption, successfully leading to the induction of ferroptosis, which was demonstrated by CLSM of the LPO probe Liperfluo. A cell-counting kit-8 (CCK-8) assay showed that LDH-based ferroptosis-targeted nanohybrids resulted in significant toxicity to HeLa, HepG2 and A549 cells. *In vivo* studies showed that the average relative tumor volume of mice treated with A@P/uLDHs was 20 times smaller than that of mice treated with Art. The above results prove that ferroptosis-targeted CDT has obvious advantages in tumor suppression and opens up a new path for the development of more efficient CDT nanomedicines.

#### Necroptosis activation-regulated CDT

As one of the most promising nanosystems, Se nanoparticles have outstanding antitumor activity and superb biocompatibility 104 and have been reported to trigger apoptotic cell death via caspase-mediated 105, Bax-dependent 106 or p53-induced 107 signaling pathways. To further improve the therapeutic index, biocompatibility and stability of Se compounds, Sonkusre *et al.* biosynthesized a novel spherical Se nanoparticle from the Bacillus licheniformis JS2 strain with an approximate diameter of 110 nm (**Figure 6D**) 40. Under the stimulation of the acidic TME of PC3 prostate cancer cells, biogenic Se nanoparticles at a concentration of 2 μg/mL enabled excessive cellular ROS generation through a Fenton-like reaction, which activated tumor necrotic factor (TNF) and interferon regulatory factor 1 (IRF1)-associated necroptosis. In addition, the presence of the necroptosis inhibitor necrostatin-1 (Nec-1) obviously increased the cell viability of the Se/Nec-1-treated group in the MTT assay. Flow cytometry analysis showed that 2 μg Se/mL resulted in an apoptosis rate of 23.79%, which was markedly higher than the 8.97% in the control group and 9.87% in the phagocytosis inhibitor cytochalasin D-treated group. Western blot analysis showed that compared to the control group, Se nanoparticles induced a significantly upregulated level of necroptosis-associated protein RIP1 after 12 h of treatment but no obvious change in RIP3 and MLKL proteins. Additionally, the necroptosis inhibitor Nec-1 had no effect on the expression levels of the above proteins. Mechanistically, Se induced RIP3/MLKL-independent necroptosis because of methylation-dependent gene silencing in PC3 cancer cells 108, while RIP1 kinase played a critical role in Se-induced necroptotic cell death. The exploration of the detailed underlying mechanism lays the foundation for designing a collaborative tumor intervention strategy based on Se nanoparticles.

ROS-based cancer CDT therapeutic exhibits great promise in suppressing tumor growth, but also suffering from the cell's inherent death resistance mechanism. The combined explorations of CDT and RCD provide certain ideas for improving the dynamic therapeutic effect of metal ions from a new perspective, and also demonstrate a series of templates for the design of a more reasonable nanochemical tumor dynamic treatment system.

### Tumor-Starvation Therapy

Cancer cells usually demonstrate stronger energy requirements than normal cells and absorb glucose, amino acids and lipids from the surrounding microenvironment at an accelerated rate to ensure sufficient substrates for ATP production and metabolism during their rapid proliferation 109. Therefore, it is promising to achieve tumor starvation treatment (TST) by directly cutting off the nutrient influx of tumor cells based on this unique feature. At present, most nanomedicine-based TSTs focus on the manufacture of glucose oxidase (GOx)-encapsulated nanosystems to catalyze glucose and O_2_ to produce gluconic acid and H_2_O_2_ to initiate tumor starvation effects 110. Utilizing the high reactivity of generated H_2_O_2_, GOx combined with other continuous redox reactions to achieve a cascade catalytic effect provides the possibility of expanding the therapeutic effect of tumor starvation.

#### Blocking autophagy-augmented TST

**Figure 7:**
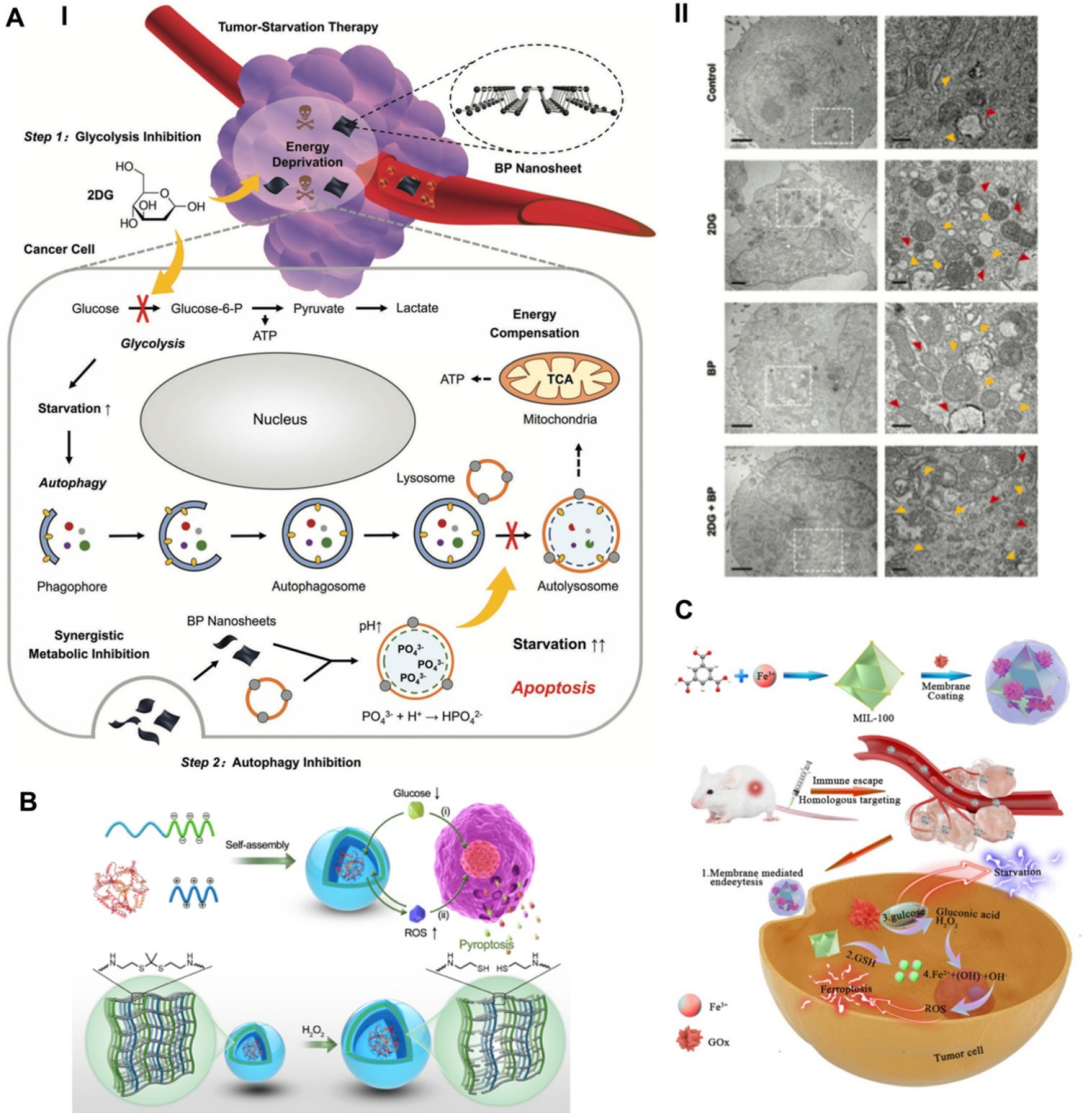
Schematic illustration of promoted tumor-starvation therapy by initiating RCD. **(A)** The mechanism of BP nanosheet-induced autophagy inhibition-improved tumor-starvation therapy of 2DG and representative bio-TEM results. Scale bars, 2 and 0.5 µm. Adapted with permission from Ref. 112, copyright 2020 WILEY-VCH Verlag GmbH & Co. KGaA, Weinheim. **(B)** Schematic illustration of the synthesis route of ROS-responsive GOD-loaded PICsomes nanoreactor and self-boosting catalytic mechanism for activating immunogenic pyroptosis-raised tumor-starvation therapy. Adapted with permission from Ref. 116, copyright 2020 WILEY-VCH Verlag GmbH & Co. KGaA, Weinheim. **(C)** Schematic illustration of preparing NMIL-100@GOx@C and the cascade catalytic processes for synergistic ferroptosis-starvation anti-tumor therapy. Adapted with permission from Ref. 115, copyright 2020 American Chemical Society.

To compensate for the lack of nutrition and metabolism, cancer cells increase self-protective autophagy levels to recycle their own cellular components, supplying additional metabolic aid pathways for rebuilding homeostasis. The inherent recycling function of autophagy can promote the survival of malignant cells under acute nutrient deficiency, thereby discounting the antitumor effect of TST 111. Taking advantage of the properties of black phosphorus (BP) to block autophagy, Yang *et al.* synthesized BP nanosheets combined with the antiglycolytic agent 2-deoxy-d-glucose (2DG) to achieve a significantly superadditive antitumor starvation treatment (**Figure 7A**) 112. Ultrathin BP nanosheets with lateral dimensions less than 200 nm were synthesized by simple liquid exfoliating technology. Due to its similar structure to glucose, the combined 2DG could be actively absorbed by glucose transporter (GLUT), thereby competitively inhibiting glucose uptake 113. Subsequently, 2DG was phosphorylated to take shape 2DG-6-phosphate by hexokinase (HK), which could not be further metabolized and noncompetitively inhibited glucose phosphorylation, thus making cancer cells prone to severe energy deficiency 114. Meanwhile, 2DG-induced downstream autophagic flux was effectively blocked by BP nanosheets based on the impairment of lysosomal functions via the neutralizing effect of BP degradation elevated alkaline phosphate anions (PO_4_^3-^) and lysosomal acid hydrogen ions. Compared with the other two single groups, the synergistic therapeutic group achieved a significantly elicited late apoptosis rate of 79.2% for A375 cells and 89.2% for HeLa cells, thus confirming the advantages of the combined therapeutic strategy. Cooperative therapy *in vivo* also achieved a higher inhibitory effect than the theoretical addition of the two monotherapies, with suppression rates of 76.97% vs. 52.32% for the A375 tumor model and 79.16% vs. 55.47% for the HeLa tumor model. Eventually, the cancer cells succumbed to therapeutic intervention and died.

#### Inducing ferroptosis-aided TST

For the purpose of amplifying the therapeutic effect of TST, Wan *et al.* proposed a synergistic ferroptosis-starvation antitumor therapy based on a cancer cell membrane-coated and GOx-loaded Fe-MOF cascade catalytic nanosystem (**Figure 7C**) 115. Fe-MOF (MIL-100) was fabricated via a hydrothermal process and applied as the iron source for ferroptosis induction and the carrier for loading GOx. Profiting from the homologous targeting and immune escape capabilities of cancer cell membranes, nanosystems were more likely to accumulate at tumor sites and be efficiently internalized by cancer cells. The high content of GSH in the TME reduced Fe^3+^ to Fe^2+^, causing the collapse of the Fe-MOF structure to free GOx and the effective delivery of iron to induce ferroptosis. GOx enables the oxidation catalytic process of glucose to generate gluconic acid and H_2_O_2_, which can consume glucose and cut off the nutrient supply of tumors 110. The excessive production of H_2_O_2_ at the tumor site provided a raw material basis for promoting the Fe-induced cascade catalytic Fenton reaction and ferroptosis therapy. Synergistically, ferroptosis induction significantly improved the sensitivity to TST treatment by sensitizing cells to apoptosis. The intervention with NMIL-100@GOx@C led to a cell survival rate of 20%, which was significantly lower than the 83.0%, 48.9%, and 30.1% of the NMIL-100@C, SiO_2_-GOx@C and NMIL-100@GOx groups, respectively. In addition, synergistic ferroptosis starvation treatment had the most efficient antitumor effect, with a tumor inhibition rate of more than 90% *in vivo*. Utilizing continuous cascade catalytic reactions to achieve a strengthened synergistic effect of tumor-targeted ferroptosis and starvation therapy represents a forward-looking strategy for highly efficient tumor treatments.

#### Initiating pyroptosis-extended TST

By applying the catalytic property of GOx, Li *et al.* synthesized a GOx-loaded and ROS-responsive therapeutic nanomaterial with self-improving catalytic glucose oxidation and pyroptosis induction functions based on polyion complex vesicles (PICsomes) (**Figure 7B**) 116. Driven by electrostatic complexation, the charged polymers were self-assembled into PICsomes through the physical mixing of poly([2-[[1-[(2-aminoethyl) thio]-1-methylethyl] thio] ethyl]-*α, β*-aspartamide) (PATK, polycation segments) and PEG-*b*-poly (*α, β*-aspartic acid) (PEG-*b*-PAsp, polyanion segments) solutions. Since the ROS cleavable linker thioketal was designed in the side chain of PATK, H_2_O_2_ caused the gradual cleavage of the ROS response linker and the stable expansion of the vesicle structure. Time-dependent DLS analysis showed that the average diameter of PICsomes increased steadily from the initial 95 nm to a large plateau after adding H_2_O_2_. The increase in vesicle volume led to a decrease in membrane cross-linking density and a significant increase in membrane permeability, which contributed to continuous improvements in the efficiency of catalyzing glucose oxidation. In addition, the expansion of vesicles resulted in the rupture and lysis of the plasma membrane of tumor cells, eventually initiating immunogenic pyroptosis. In particular, PICsomes enabled the protection of GOx to maintain its long-term cytocidal activity. The induction of pyroptosis significantly enhanced the immunogenic response and thus synergistically promoted the tumor suppressive effect of TST. The self-boosting catalytic nanoreactor exhibited an advantageous intervention effect on 4T1 breast cancer cells via pyroptosis-extended glucose starvation.

TST induces tumor cell starvation by directly cutting off the supply of energy and nutrients, which can trigger cell apoptosis. On the basis of TST, sensitized tumor therapy can be achieved by inhibiting autophagy, inducing ferrptosis and targeting pyroptosis. It is expected that this combined strategy of simultaneously blocking nutrient supply pathways and regulating RCD may be of great significance to the design of tumor-specific nanotherapies in the future.

### Tumor-Radio/Chemotherapy

As the most commonly used front-line methods in the clinic, radiotherapy (RT) and chemotherapy (CT) play an extremely significant role in inhibiting tumor progression and prolonging the life of tumor patients. At the same time, radio/chemotherapy (RCT)-induced severe side effects and drug resistance are also practical problems that must be addressed 117. The advancement and development of nanomedicine provides technical support for targeted drug delivery, increases radiation accumulation and reduces toxic side effects. In addition, advanced nanotechnology can achieve high-efficiency radiochemical tumor treatment based on multiple RCD interventions and synergistic enhancement 118.

#### Autophagy modulation-supported RCT

RCT usually activates protective autophagy in cancer cells, which leads to elevated therapeutic resistance and compromises its inhibitory effects 59. However, sustained and full autophagy may promote cell death due to excessive self-digestion. Based on this, Hu *et al.* investigated the possibility of zinc oxide nanoparticle (ZON)-elevated autophagy to synergistically enhance the effect of tumor CT 119. ZON-elicited degradative autophagy contributed to promoting the killing effect of tumor cells by accelerating intracellular lysis, releasing ZONs and generating ROS. More importantly, combinational ZON intervention with doxorubicin (Dox) resulted in sensitized chemotherapeutic effects on drug-resistant cancer cells due to synergistic dual pro-death autophagy activation. Systematic experimental results demonstrated that the antitumor therapeutic effect of combined administration significantly exceeded those of ZONs or Dox alone, thus confirming the critical role of autophagy modulation in potentiating chemotherapeutic-mediated cancer cell death.

**Figure 8:**
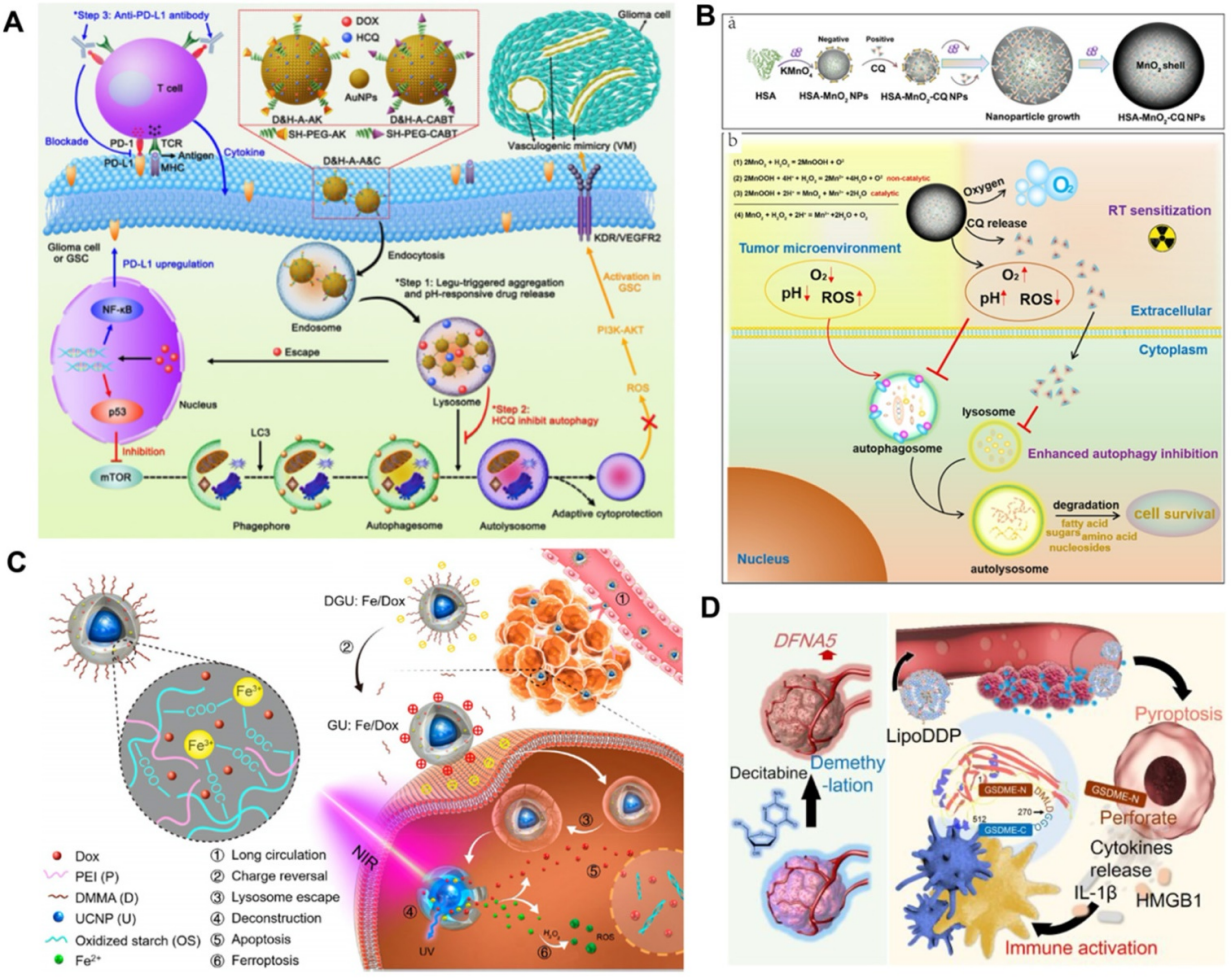
Schematic illustration of combinational RCD for synergistic improved tumor killing of radio/chemotherapy. **(A)** Schematic diagram of the antitumor therapy of AuNPs-enabled chemotherapy and autophagy inhibition with PD-L1 checkpoint blockade. Adapted with permission from Ref. 120, copyright 2020 American Chemical Society. **(B)** The preparation routine of HSA-MnO_2_-CQ nanoparticles and anti-tumor mechanism by autophagy inhibition combined radiation therapy. Adapted with permission from Ref. 122, copyright 2020 Ivyspring International Publisher. **(C)** Schematic illustration of the Fe^3+^ cross-linked structure of nanolongan carrier co-loading UCNP and Dox, and dual RCD anti-tumor mechanism of chemotherapy. Adapted with permission from Ref. 123, copyright 2019 American Chemical Society. **(D)** Schematic illustration of activating epigenetics-based pyroptosis to enhance immunological anti-tumor effect of chemotherapy. Adapted with permission from Ref. 126, copyright 2019 American Chemical Society.

In contrast to activating autophagy to potentiate CT, inhibiting autophagy upregulated by acidic TME can also sensitize the tumor suppressive effect of RCT. Ruan *et al.* proposed an enhanced tumor CT strategy combined with autophagy inhibition based on the multi-functional gold (Au) nanoparticles that co-loading with Dox and HCQ 120 (**Figure 8A**). The modified Au nanoparticles with the legumain-responsive property could improve the accumulation of Dox at tumor site overexpressing legumain and promote tumor inhibition. However, chemodrug Dox could also induce pro-survival autophagic mechanism to discount CT by recycling nutrients and formatting vasculogenic mimicry 121. Fortunately, the co-delivered HCQ can synergistically promoted the inhibitory effect of CT by blocking the fusion of autophagolysosomes and autophagosomes. In addition, the combination with anti-PD-L1 further heightened the antitumor effect of CT. Thus, the strategy of regulating autophagy presents a valuable way to develop more predominant tumor CT. Considering the high affinity between positively charged CQ and negatively charged MnO_2_, Lin *et al.* fabricated a human serum albumin (HSA)-based nanosystem by integrating MnO_2_ and CQ (HSA-MnO_2_-CQ nanoparticles, HMCQ NPs) (**Figure 8B**) 122. HMCQ NPs were synthesized by first obtaining HM NPs through the reduction of KMnO_4_, followed by deposition on HSA molecules and the subsequent acquisition of HMCQ NPs via CQ deposition on HM NPs. HMCQ NPs effectively produced O_2_ and increased the pH to change the TME by reacting with H^+^/H_2_O_2_ and released CQ to block hypoxia-triggered autophagic flux. Inhibition of protective autophagy blocked the self-adaptive supplementation of energy by tumor cells and reduced the resistance of cells to RT, thereby enhancing the inhibitory effect of RT. Finally, remarkably sensitized RT antineoplastic effects on bladder tumors were achieved by cooperatively regulating the abnormal TME and inhibiting prosurvival autophagic flux.

#### Ferroptosis regulation-facilitated CT

Generally, CT not only triggers apoptotic cancer cell death by invading the nucleus and damaging DNA but also induces serious side effects and drug resistance. Suffering from the disappointing therapeutic effect of CT, Bao *et al.* proposed a combined anticancer CT strategy by inducing dual ferroptosis/apoptosis of cancer cells to promote tumor suppression (**Figure 8C**) 123. They designed a novel nanolongan drug delivery system with a representative composition of one core in one gel nanoparticle. The nanolongan system DGU was synthesized by encapsulating upconversion nanoparticles (UCNPs) and Dox with oxidized starch-based gel nanoparticles, sequential cross-linking with Fe^3+^ ions, and further decorating with polyethylenimine (PEI) and 2,3-dimethylmaleic anhydride (DMMA). Negatively charged DMMA promoted the targeted aggregation of nanoparticles to the tumor site. Subsequently, under the stimulation of an acidic TME, the nanolongan reversed to a positive charge, which facilitated the escape of lysosomes through the proton sponge effect. Under NIR radiation, UCNPs enabled the reduction of Fe^3+^ to Fe^2+^ and the release of Dox, thereby promoting Fe^2+^-induced ferroptosis and Dox-triggered apoptosis. Dual ferroptosis/apoptosis RCD activation effectively sensitizes tumor cells to the killing effect of chemotherapy on drug resistance. Multifunctional nanolongan achieved exceptional antitumor therapeutic effects and could act as a safe and efficient modality of ferroptosis/apoptosis synergistic antitumor therapy.

#### Pyroptosis triggering-enhanced CT

Several chemotherapeutic drugs initiate caspase-3-induced apoptosis to kill cancer cells, which also lays the foundation for mediating the pyroptosis process based on gasdermin E (GSDME) translated by the deafness autosomal dominant 5 (DFNA5) gene 124. However, most tumor cells lack the key protein GSDME in caspase-3-mediated pyroptosis due to hypermethylation of the DFNA5 gene 125. In response to this problem, Fan *et al.* designed an advantageous tumor suppression strategy by triggering pyroptosis of cancer cells based on the synergistic effect of CT and DNA demethylation (**Figure 8D**) 126. They first used epigenetics to pretreat tumors with the DNA methyltransferase decitabine (DAC) to achieve demethylation of the DFNA5 gene in tumor cells. Subsequently, tumor-targeted nanoliposomes loaded with the chemotherapeutic drug cisplatin (LipoDDP) were synthesized by the film ultrasonic method and utilized to activate the caspase-3 pathway and induce pyroptosis in tumor cells. Impressively, epigenetics-based chemotherapeutics not only reversed GSDME silencing and promoted the occurrence of pyroptosis but also triggered an effective immune response to prevent tumor recurrence and metastasis. The pyroptosis-based CT strategy obviously improved tumor suppression effects and offered remarkable insights into tumor immunotherapy.

Since the effect of RCT-induced apoptotic death is usually limited by the inherent or evolved RCT resistance of tumor cells, the combined intervention of RCD and RCT provides a promising way to significantly improve the efficacy of anti-cancer treatments by sensitizing radiotherapy and chemotherapy.

## Concluding Remarks

Due to the inherent or evolved apoptosis resistance mechanism, cancer cells often exhibit special defects in cell death execution. Therefore, an increasing number of studies have focused on targeting the nonapoptotic routes of RCD based on advanced and rapidly developing nanobiotechnology to improve cancer therapeutic efficiency. Recent progress has proven that RCD makes great contributions to synergistically enhanced tumor nanotherapies such as PDT, SDT, PTT, CDT, and immunotherapy (**Table 1**). Compared with single apoptosis induction, triggering dual RCDs, such as ferroptosis/apoptosis, pyroptosis/apoptosis, or necroptosis/apoptosis, may significantly decrease apoptosis resistance and make cancer cells more vulnerable to external nanotherapeutics. In this regard, the progress review highlights the state-of-art developments in RCD-based nanomedicine within the biomedical oncology field and demonstrates its great therapeutic potential as a novel way to combat apoptosis-resistant tumors.

## Future Perspectives

Although substantial progress has resulted in satisfactory anticancer therapeutic efficiency, targeting nonapoptotic RCD routes is still in the preliminary exploration stage in biomedical oncology fields, as several key issues and challenges must be addressed to further strengthen the potential clinical transformation of this research. In the future, the following aspects should be taken into consideration: (1) Novel nanomaterials should be designed to achieve more efficient synergistic RCD anticancer therapies, and the physicochemical properties of the nanomaterials should be explored, such as the size, shape, stiffness, chemical composition and surface charge, to determine the effect on the induction of different RCD types. (2) The specificity of inducing RCD with several nanomaterials must continue to be explored in preclinical and clinical cancer environments to achieve precise understanding and control of its potential side effects. (3) The role of the cellular setting in RCD stimulation and that of nanomaterial-mediated signaling pathway alterations in critical cellular functions must be evaluated to clarify the mutual influence of the TME and nanomaterials in RCD. (4) Other synergistic combinations of RCD must be explored, and the probability and factors of conversion between different RCD pathways should be exploited to achieve the best tumor treatment strategy. (5) As a stepping stone to clinical translation, more detailed mechanistic insights are still needed, as well as real-time imaging studies, to evaluate the utility of RCD in combating apoptosis-resistant cancers.

The emerging RCD-based nanobiomedical strategy for sensitized tumor therapy not only has the advantages of higher efficiency and increased safety but also overcomes the inherent apoptosis resistance characteristics of cancer cells. Despite certain unresolved issues, the as-demonstrated fascinating antitumor performance of RCD-assisted cancer nanotherapeutics, which is expected to become a burgeoning research hotspot in the field of cancer nanomedicine, illustrates a strong potential for clinical translation and the benefit of patients.

## Author Contributions

Q.H. Zeng and X.Y. Ma contributed equally to this work. Q.H. Zeng, X.Y. Ma and Y.M.H. Song collected the related paper and drafted the manuscript. Q.H. Zeng and Q.Q. Chen prepared the figures. X.Y. Ma, Q.L. Jiao and L.Q. Zhou helped to revise the manuscript. Q.L. Jiao and L.Q. Zhou designed the review and provided supervision.

## Figures and Tables

**Table 1:** Summary of RCD-based biomedicines for cooperative cancer nanotherapy

Categories	Auxiliary therapies	RCD inductions	Nanomaterials	Tumor cells	Synergistic effects	References
Autophagy	PDT	3BP	CD-Ce6-3BP	MNNG/HOS osteosarcoma cells (BALB/c)	Overcome autophagy-induced cancer cell resistance of PDT against ROS damage	51
	SDT	3-MA	PpIX/3-MA@Lip	MCF7 breast cancer cells (BALB/c)	Improve the anti-tumor effect of SDT by blocking cell self-protective autophagy	59
	PTT	3-MA/CQ	TNP-1	4T1 and MCF7 breast cancer cells (BALB/c)	Manipulate autophagy to achieve effective photothermal effect of mild PTT	77
	Immunotherapy	STF-62247	ASN	CT26 Colon cancer cells (BALB/c)	On-demand autophagy cascade amplification to boost anti-tumor immunotherapy	74
	CDT	CQ	MIL-88B	A357 and HeLa cells (BALB/c)	Cut off the self-protection pathway and induce amplified oxidative damage	99
	TST	BP	BP-2DG	A357 and HeLa cells (BALB/c)	Block the recycling of nutrients to increase tumor starvation effect induced by TST	112
	CT	ZONs	ZONs-Dox	T24 bladder cancer cells (BALB/c)	Dual autophagy activation to potentiate chemotherapeutic-mediated cancer cell death	119
	RT	CQ	HMCQ	4T1 and MCF7 breast cancer cells (BALB/c)	Inhibit TME-upregulated autophagy to sensitize tumor suppression effect of RT	122
Ferroptosis	PDT	SRF	SFT-MB	4T1 breast cancer cells (BALB/c)	Contribute to improving the sensitivity of apoptosis-induction in PDT	55
	SDT	Ferumoxytol	Lipo-PpIX@Ferumoxytol	4T1 breast cancer cells (BALB/c)	Target ferroptosis for sensitizing ROS-resistant tumors to pro-apoptotic SDT	61
	PTT	F4TCNQ	TMB-F4TCNQ	4T1 and HeLa cells (BALB/c)	GSH depletion-induced ferroptosis increase the sensitivity of PTT-mediated cell death	79
	Immunotherapy	RSL-3	BNP@R	4T1 and B16-F10 cells (C57BL/6)	Ferroptosis-induced lipid peroxide promoted the phagocytosis of the tumor by DCs	95
	CDT	LDH	A@P/uLDH	Hela, HepG2 and A549 cells (BALB/c)	Ferroptosis targeting sensitizes CDT-induced cell apoptosis	103
	TST	Fe-MOF	Fe-MOF@GOx	4T1 breast cancer cells (BALB/c)	GOx starvation effect amplify the Fenton reaction of Fe in cascade	115
	CT	Fe irons	DGU	4T1 and HeLa cells (BALB/c)	Overcome the drug resistance of CT and enhance CT-induced cell apoptosis	123
Pyroptosis	PDT	TBD-3C	TBD	4T1, HeLa and C6 cells (NA)	Increase the PDT efficiency by pyroptosis-triggered self-immune response	28
	Immunotherapy	Ca irons	BNP	4T1 breast cancer cells (BALB/c)	Activated pyroptosis promoted DCs maturation and systemic immune response	30
	CDT	Na irons	PNSO	4T1 and CT26 cells (BALB/c)	Regulate immunosuppressed TME and trigger anti-tumor immune responses of CDT	102
	TST	PATK	PICsomes	4T1 and MB231 cells (NA)	Induced immunogenic pyroptosis to improve the efficiency of catalytic TST	116
	CT	DAC	LipoDDP	4T1 and CT26 cells (BALB/c)	Enhance the immunological effect of CT by epigenetics-based pyroptosis	126
Necroptosis	SDT	PFP	NB	CT26 Colon cancer cell (BALB/c)	Induce necroptosis to improve SDT-activated anti-tumor immune responses	66
	PTT	CuS-NiS_2_	CuS-NiS_2_	AGS and MKN-45 gastric cancer cell (BALB/c)	Dual induction of apoptosis/necroptosis to heighten the photothermal effect	81
	CDT	SeNP	SeNP	PC3 prostate cancer cells (NA)	Improve the oxidative stress efficiency of CDT by inducing multiple RCDs	40

Abbreviations: RCD, regulated cell death; PDT, photodynamic therapy; SDT, sonodynamic therapy; PTT, photothermal therapy; CDT, chemodynamic therapy; TST, tumor-starving therapy; CT, chemotherapy; RT, radiotherapy; 3BP, 3-bromopyruvate; CD, α-cyclodextrin; Ce6, chlorin e6; 3-MA, 3-methyladenine; CQ, chloroquine; TNP-1, tetrapod nanoparticle-1; ASN, autophagy sensitive nanoparticle; BP, black phosphorus; 2DG, 2-Deoxy-d-glucose; ZONs, zinc oxide nanoparticles; HMCQ, HSA-MnO_2_-CQ; SRF, sorafenib; MB, methylene blue; SFT-MB, SRF@FeIIITA-MB; BNP, biomimetic nanoparticle; LDH, Layered double hydroxide; A@P: PEG-encapsulated Artemisinin; PNSO, phospholipid modified sodium persulfate; PATK, poly([2-[[1-[(2-aminoethyl) thio]-1-methylethyl] thio] ethyl]-α, β-aspartamide); PICsomes, polyion complex vesicles; DAC, decitabine; PFP, perfluoropentane; NB, nanobubble; SeNP, selenium nanoparticle.

## Abbreviations

NCCD: Nomenclature Committee on Cell Death
ACD: accidental cell death
RCD: regulated cell death
PDT: photodynamic therapy
SDT: sonodynamic therapy
PTT: photothermal therapy
CDT: chemodynamic therapy
TST: tumor-starving therapy
CT: chemotherapy
RT: radiotherapy
NADPH: nicotinamide adenine dinucleotide phosphate
GSH: glutathione
GPX4: glutathione peroxidase 4
ROS: reactive oxygen species
GSDMD: gasdermin D
LDH: lactate dehydrogenase
3BP: 3-bromopyruvate
CD: α-cyclodextrin
Ce6: chlorin e6
3-MA: 3-methyladenine
CQ: chloroquine
TNP-1: tetrapod nanoparticle-1
ASN: autophagy sensitive nanoparticle
BP: black phosphorus
2DG: 2-Deoxy-d-glucose
ZONs: zinc oxide nanoparticles
HMCQ: HSA-MnO_2_-CQ
SRF: sorafenib
MB: methylene blue
SFT-MB: SRF@FeIIITA-MB
BNP: biomimetic nanoparticle
LDH: Layered double hydroxide
A@P: PEG-encapsulated Artemisinin
PNSO: phospholipid modified sodium persulfate
PATK: poly([2-[[1-[(2-aminoethyl) thio]-1-methylethyl] thio] ethyl]-α, β-aspartamide)
PICsomes: polyion complex vesicles
DAC: decitabine
PFP: perfluoropentane
NB: nanobubble
SeNP: selenium nanoparticle
